# Sustained Inhibition of Maize Seed‐Borne *Fusarium* Using a *Bacillus*‐Dominated Rhizospheric Stable Core Microbiota with Unique Cooperative Patterns

**DOI:** 10.1002/advs.202205215

**Published:** 2022-12-18

**Authors:** Weibing Xun, Yi Ren, He Yan, Aiyuan Ma, Zihao Liu, Lingling Wang, Nan Zhang, Zhihui Xu, Youzhi Miao, Haichao Feng, Qirong Shen, Ruifu Zhang

**Affiliations:** ^1^ Jiangsu Provincial Key Lab of Solid Organic Waste Utilization Jiangsu Collaborative Innovation Center of Solid Organic Wastes Nanjing Agricultural University Nanjing Jiangsu 210095 P. R. China; ^2^ Key Laboratory of Microbial Resources Collection and Preservation Ministry of Agriculture Institute of Agricultural Resources and Regional Planning Chinese Academy of Agricultural Sciences Beijing 100081 P. R. China

**Keywords:** core rhizobacteria, microbial cooperation, seed‐borne pathogens, stable and high‐performance, synthetic microbiota

## Abstract

Seed‐borne pathogens can inhabit the rhizosphere and infect the plant after germination. The rhizosphere microbiome plays critical roles in defending against seed‐borne pathogens. However, the assembly of a core rhizosphere microbiome to suppress seed‐borne pathogens is unknown. Here, the root‐associated microbiome is infested with seed‐borne *Fusarium* in sterile environment, while the root‐associated microbiome is not infested when it interacts with the native soil microbiome across maize cultivars, suggesting that a core rhizosphere microbiome assembles to suppress seed‐borne *Fusarium*. Two strategies of progressive dilution and rhizodepositional attraction are applied to identify the core rhizobacteria. A synthetic microbiota (SynM) is constructed using the isolates of the core rhizobacteria and optimized according to superior community stability and *Fusarium*‐suppression capability, which surpasses the single strain and randomly formed microbiota. The optimized SynM (OptSynM) presents a distinctive cooperative pattern in which a key strain harbors the *Fusarium* suppression function by synthesizing the antagonistic substance fengycin, while other members intensify the functional performance by promoting the growth and the expression of the antagonistic and plant‐growth‐promoting related genes of the key strain. This study demonstrates innovative approaches to construct stable and minimal microbiota for sustainable agriculture and proposes a unique cooperative pattern to sustain community stability and functionality.

## Graphical abstract

The authors identify a core rhizosphere microbiome assembled to suppress the seed‐borne *Fusarium*. They construct a simplified and stable synthetic microbiota that present a distinctive cooperative pattern to inhibit seed‐borne *Fusarium* using culturable core bacterial strains. The simplified microbiota is capable to suppress seed‐borne *Fusarium* across all the collected commercial maize cultivars under microcosm cultivation conditions in a sustainable manner.

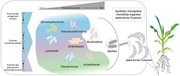

## 1 Introduction

Plant diseases caused by soil‐ or seed‐borne pathogens are serious threats to crop production and food security worldwide.^[^ ^1^ ^]^ Compared with soil‐borne pathogens, seed‐borne pathogens are able to inhabit the integument, endosperm, or embryo of plant seeds, allowing for widespread dissemination through contaminated seed banks.^[^ ^2^, ^3^ ^]^ This widespread dissemination is the case for *Fusarium*, a genus of filamentous fungi encompassing many globally important and widespread plant pathogens^[^ ^4^ ^]^ that have established seed‐based transmission modes.^[^ ^5^, ^6^ ^]^ Seed surface disinfection may adversely affect seed germination and does not guarantee the inactivation of seed‐borne pathogens, as a low level of contamination is sufficient to induce efficient plant colonization,^[^ ^7^ ^]^ resulting in high rates of disease incidence.^[^ ^8^ ^]^ Plants have developed responses that include activation of their innate immune system,^[^ ^9^ ^]^ systemic acquired resistance,^[^ ^10^ ^]^ and induced systemic resistance^[^ ^11^ ^]^ against pathogen invasion. However, the ability of the plant to resist infection by seed‐borne *Fusarium* was rather weak in a soil‐less cultivation,^[^ ^8^, ^12^ ^]^ suggesting that a native soil microbiome plays an important role in the suppression of seed‐borne pathogens.^[^ ^13^ ^]^

Plants have the ability to recruit beneficial microbes from the soil microbiome,^[^ ^14^, ^15^ ^]^ thereby enhancing their resistance to pathogen or insect attack.^[^ ^16^, ^17^ ^]^ The importance of root‐associated microbes, which are largely influenced by root exudates, has been increasingly recognized in the promotion of plant growth and health.^[^ ^18^, ^19^ ^]^ A stable suppression effect within the soil ecosystem is more likely due to a cohort of microbes that possess antagonistic properties or induce competition for space or nutrients against the pathogen.^[^ ^20^ ^]^ To this end, research has been conducted using synthetic microbiotas (SynMs) to investigate plant–microbe interactions, although their focus is principally on function^[^ ^21^, ^22^, ^23^ ^]^ rather than the redundancy or stability of the microbiota.^[^ ^24^ ^]^ Therefore, a current research challenge is the construction of a SynM to assess both the functional efficiency and compositional stability when challenged by a pathogen. This SynM would be expected to reveal the intrinsic interactions among the individual populations that correspond to pathogen suppression.

**Figure 1:**
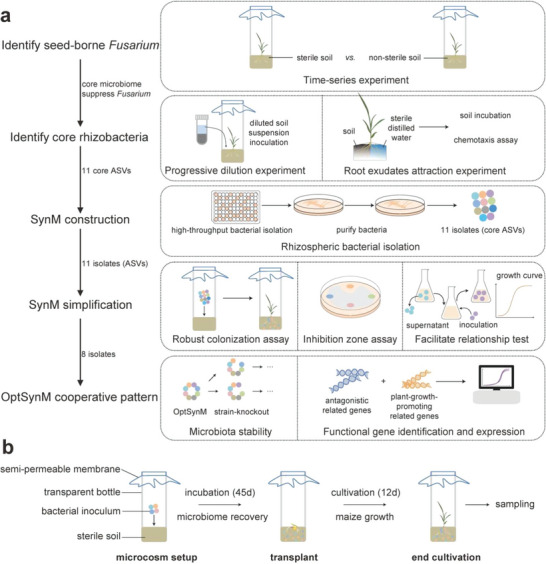
An overview of the experimental procedure in this study. a) Experimental procedure. b) The cultivation microcosm setup, cultivation, and sampling.

In this study (**Figure** **1**a), we showed that seed‐borne *Fusarium* is widely distributed among maize across various cultivars. The maize rhizosphere and root endosphere microbiomes were infested with *Fusarium* when grown in sterile soil, whereas the seed‐borne *Fusarium* was suppressed when interacting with a native soil microbiome in the non‐sterile soil. A two‐pronged innovative approach was developed to identify the core rhizosphere bacteria that were associated with seed‐borne *Fusarium* suppression, coupled with the isolation of the identified strains to construct a minimal and stable SynM that exhibits sustainable disease suppression and maize growth promotion. Finally, we revealed that this SynM presented a distinctive intrinsic cooperative pattern, in which a key strain played a major role in suppressing seed‐borne *Fusarium* and promoting maize growth, while other members maintained the stability of the microbiota and intensified the functional performance of the key strain.

## 2 Results

### 2.1 Seed‐Borne *Fusarium* Is Prevalent in Maize Cultivars

**Figure 2:**
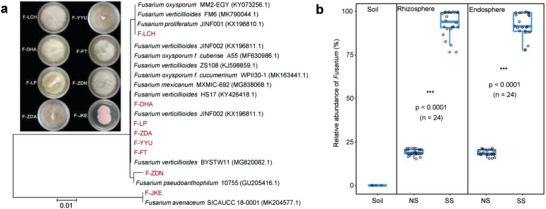
Seed‐borne *Fusarium* in maize cultivars. a) The seed‐borne fungal strains isolated from eight commercial maize cultivars and an unrooted phylogenetic tree of seed‐borne *Fusarium* strains based on ITS sequences using the neighbor‐joining method. The abbreviations of *Fusarium* strains were named after the maize varieties; see details in Table S1, Supporting Information. b) The relative abundance of *Fusarium* in bulk soil, rhizosphere, and root endosphere fungal communities when cultivated in both non‐sterile and sterile soils. NS: non‐sterile soil. SS: sterile soil. Asterisks indicate significance: ***p <0.001 based on two‐sided *t*‐test (*n* = 24).

When surface‐disinfected maize seeds were germinated and cultivated in Murashige and Skoog (MS) medium, the roots of the seedlings were invariably covered by visible fungal mycelia (Figure S1a, Supporting Information). We tested eight maize cultivars collected from different major planting areas of China and observed similar phenomena (Table S1 and Figure S1b, Supporting Information), indicating the ubiquity of maize seed‐borne fungi. These dominant fungi were isolated from all cultivars and identified to be *Fusarium* spp. based on their internal transcribed spacer (ITS) sequences (**Figure** **2**a). We then cultivated eight maize cultivars in both non‐sterile and sterile soils and observed that the seeds became infested by the seed‐borne *Fusarium* and that the maize root and stem base were covered by *Fusarium* mycelium in sterile soil. Amplicon sequencing was applied to assay the rhizosphere and root endosphere fungal community composition, and further analysis indicated that seed‐borne *Fusarium* was dominant (relative abundance, RA: 83.8–99.1%) when cultivated in sterile soil but lower (RA: 16.7–19.8%) in non‐sterile soil (Figure 2b and Figure S1c, Supporting Information). At the species (amplicon sequence variants, ASV) level of taxonomic resolution, we confirmed that the infested *Fusarium* species generated from amplicon sequencing were consistent with the fungal isolates from each cultivar.

To confirm whether seed‐borne *Fusarium* can dominate a “native” root‐associated fungal community in a sterile environment, we established a time‐series longitudinal dense sampling experiment by cultivating one representative maize cultivar (Fengtian843; FT) in both non‐sterile and sterile soils. For all of the root‐associated fungal communities, we observed that seed‐borne *Fusarium* was the dominant species (RA > 82.8%) during the entire survey period (Figure S2, Supporting Information). This *Fusarium* infestation inhibited maize growth by decreasing plant height and shoot fresh weight while increasing the activities of the plant stress tolerance‐related antioxidant enzymes peroxidase (POD), superoxide dismutase (SOD), and catalase (CAT) (Figure S2g, Supporting Information).

Given that seed‐borne *Fusarium* dominated the root‐associated communities from germination to 3‐week‐old seedlings in sterile soil but was suppressed in non‐sterile soil, we classified a maize seedling as a *Fusarium*‐infected seedling when the relative abundance of *Fusarium* was above 80% in the root‐associated community and as a *Fusarium*‐suppressed seedling when it was below 20%.

### 2.2 Identification of Core Rhizobacteria for Seed‐Borne *Fusarium* Suppression

The finding of a general suppression of seed‐borne *Fusarium* in the non‐sterile soils suggests that inhibitory functions derived from the root‐associated microbiome may exist. Consequently, two strategies were applied to identify the core microbiome that could then be isolated to construct a synthetic *Fusarium*‐suppressive microbiota.

**Figure 3:**
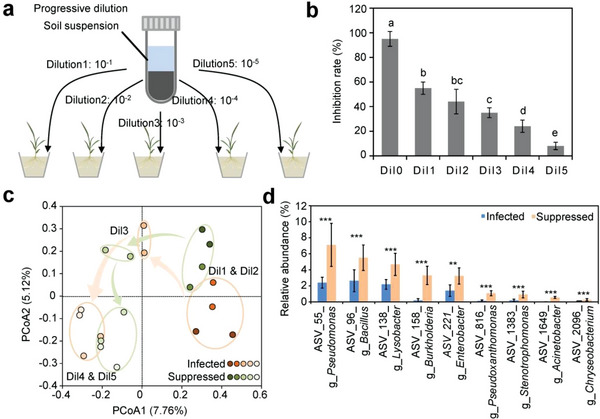
Identification of the core rhizobacteria using progressive dilution. a) A sketch map of the progressive dilution experiment. b) The seed‐borne *Fusarium* inhibition rate in the progressive dilution experiment. Dil0 indicates the non‐sterile soil. Dil1 to Dil5 indicate the dilution level from 10^−1^ to 10^−5^. c) PCoA of the rhizosphere bacterial communities in the progressive dilution experiment. Different letters above bars indicate significant differences (*p* < 0.05) according to Duncan's multiple comparison. d) The relative abundance of the shared indicator taxa (see details in Figure S3e, Supporting Information) in the rhizosphere bacterial communities of *Fusarium*‐suppressed seedlings. Asterisks indicate significance: **p <0.01 and ***p <0.001 based on two‐sided *t*‐test. Error bars represent standard deviations (*n* = 2 and 10 in [b] and [d], respectively).

The first strategy was to establish a progressive dilution experiment that would gradually remove the low abundance taxa (**Figure** **3**a). We hypothesized that the seed‐borne *Fusarium* cannot be suppressed when the core beneficial species are absent by increasing dilution intensity. As expected, the non‐sterile soil maintained a high disease inhibition rate of 95%, which was dramatically reduced to 8% at the highest dilution of 10^−5^ (Figure 3b). To understand whether this *Fusarium* suppression is governed by the rhizosphere or root endosphere community, we used 16S ribosomal RNA amplicon sequencing to assay the community composition (Figure 3c and Figure S3a, Supporting Information). Compared to the non‐significantly changed root endosphere community, we observed a dramatic loss of rhizosphere bacterial richness and increased community dissimilarity with increasing dilution intensity (Figure S3b,c, Supporting Information), which induced a significant decrease in the seed‐borne *Fusarium* inhibition rate. This result suggested that *Fusarium* suppression was attributed mainly to rhizosphere microbes. To discern the bacterial indicator taxa that may contribute to *Fusarium* suppression, we applied partial least squares discrimination analysis (PLS‐DA) and random‐forest (RF) machine learning methods (Figure S3d,e, Supporting Information). Five repeated 10‐fold cross‐validations showed an optimal number of indicator taxa at 12. Among these shared indicator taxa, we identified 9 enriched ASVs in the *Fusarium*‐suppressed group (Figure 3d).

**Figure 4:**
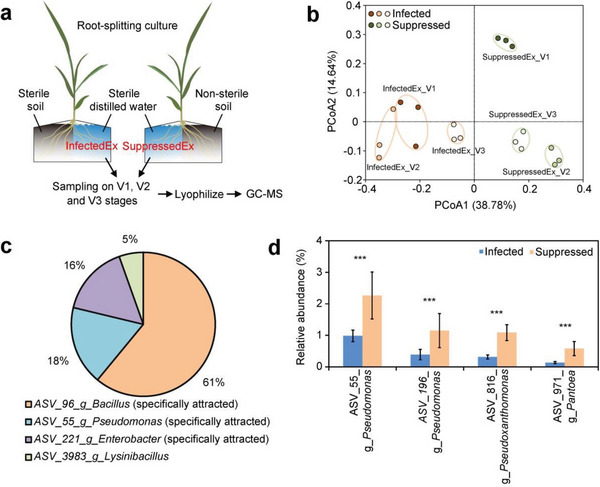
Identification of the core rhizobacteria using root exudate attraction. a) A sketch map of the root‐splitting device used to collect the root exudates. b) PCoA of the root exudate composition of *Fusarium*‐infected (InfectedEx from sterile soil) and *Fusarium*‐suppressed (SuppressedEx from non‐sterile soil) seedlings at the one‐leaf (V1), two‐leaf (V2), and three‐leaf (V3) stages. The InfectedEx were compositionally different (ANOSIM *R*‐value = 0.715, *p* < 0.001) from SuppressedEx. c) The ASVs (percentages shown in the pie chart) attracted in the chemotaxis assay. Three of the four detected ASVs were specifically attracted by the SuppressedEx compounds. d) The relative abundance of four abundant (RA > 0.5%) ASVs that were simultaneously enriched by the SuppressedEx compounds (T_SuppressedEx_All and T_SuppressedEx_V2, see details in Table S3 and Figure S4, Supporting Information) in the soil incubation assay. Asterisks indicate significance: **p <0.01 and ***p <0.001 based on two‐sided *t*‐test. Error bars represent standard deviations (*n* = 16).

Another strategy was to elucidate the contribution of root exudates since the recruitment of beneficial bacteria from the bulk soil to the rhizosphere may largely be the result of rhizodepositional attraction. A root‐splitting device was applied to collect the root exudates of *Fusarium*‐infected (InfectedEx from sterile soil) and *Fusarium*‐suppressed (SuppressedEx from non‐sterile soil) maize seedlings at the one‐leaf (V1), two‐leaf (V2), and three‐leaf (V3) growth stages (**Figure** **4**a). Sugars, organic acids, and alcohols were the most abundant compounds (Figure S4a–d and Table S2, Supporting Information). More sugars but fewer organic acids and alcohols were produced in SuppressedEx than in InfectedEx. Considering that the root exudates at the V2 stage illustrated the most profound compositional differences between InfectedEx and SuppressedEx, we conducted a chemotaxis and a soil incubation assay using the mixture of the identified typical compounds (Figure 4b and Table S3, Supporting Information) to clarify their recruiting effects on soil microbes. We detected four ASVs in the chemotaxis assay, in which three ASVs were specifically attracted by the SuppressedEx compounds (Figure 4c and Figure S4e, Supporting Information). Soil incubation demonstrated that four abundant ASVs (RA > 0.5%) were enriched by SuppressedEx compounds while two ASVs were enriched by InfectedEx compounds (Figure 4d and Figure S4f,g, Supporting Information). Therefore, we identified 9, 3, and 4 enriched ASVs in the progressive dilution, chemotaxis, and soil incubation assays, respectively, resulting in 11 nonredundant core ASVs that may contribute to *Fusarium* suppression (Table S4, Supporting Information).

### 2.3 Synthetic Microbiota Construction and Simplification

To construct a simplified and stable SynM and verify its inhibitory effect on maize seed‐borne *Fusarium*, we isolated 2156 bacterial isolates containing 237 unique strains, from the rhizosphere of *Fusarium*‐suppressed seedlings (Figure S5a, Supporting Information) and constructed an 11‐strain SynM using the 11 core ASVs (Table S4, Supporting Information). We also constructed multiple SynMs in parallel as controls (see details in the Experimental Section), which contained: 1) different ASVs from the same genera of the 11 strains in SynM, 2) randomly selected enriched ASVs in *Fusarium*‐suppressed seedlings, 3) randomly selected depleted ASVs in *Fusarium*‐suppressed seedlings, and 4) randomly selected ASVs from the unique strains. The rationally designed 11‐strain SynM inoculation presented the greatest inhibition rate of 84.5% in sterile soil (Figure S5b, Supporting Information), suggesting that our strategy based on the core rhizosphere bacteria is valid in designing an effective SynM.

**Figure 5:**
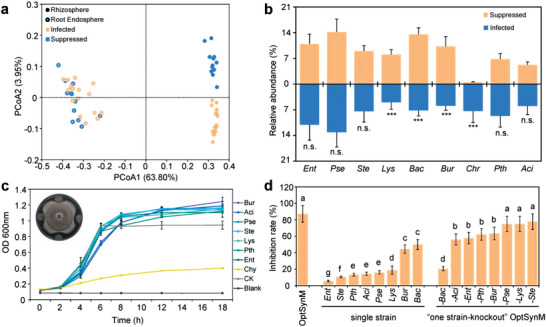
Synthetic microbiota (SynM) construction and simplification. a) PCoA of the root‐associated bacterial communities of *Fusarium*‐infected and *Fusarium*‐suppressed groups after inoculation with SynM. The rhizosphere communities were significantly different (ANOSIM *R*‐value = 0.882, *p* < 0.001), while the root endosphere communities showed non‐significant differences (ANOSIM *R*‐value = 0.204, *p* = 0.06) between the *Fusarium‐*infected and *Fusarium‐*suppressed samples. b) The relative abundance of the nine detected ASVs in rhizosphere communities of the *Fusarium*‐infected and *Fusarium*‐suppressed groups. Error bars represent standard deviations (*n* = 12 and 16 for *Fusarium*‐infected and *Fusarium*‐suppressed groups, respectively). Asterisks indicate significance: ***p <0.001 based on two‐sided *t*‐test, n.s: not significant (*p* > 0.05). c) The growth curves of *Bac* affected by the supernatants of eight strains of *Pse*, *Lys*, *Bur*, *Ent*, *Pth*, *Ste*, *Aci*, and *Chr*. The inset shows the direct antagonistic activity of *Bac* against *Fusarium* using inhibition zone assay on a plate. d) The seed‐borne *Fusarium* inhibition rate of the 8‐strain OptSynM, “one strain‐knockout” OptSynM and single strain. OptSynM, the 8‐strain OptSynM. −*Bac*, −*Aci*, −*Ent*, −*Pth*, −*Bur*, −*Pse*, −*Lys*, and −*Ste* represent the “one strain‐knockout” OptSynMs obtained by removing each indicated strain from the 8‐strain OptSynM. Different letters above bars indicate significant differences (*p* < 0.05) according to Duncan's multiple comparison. Error bars represent standard deviations (*n* = 10 and 3 in [c] and [d], respectively).

To simplify the rationally designed 11‐strain SynM, we further analyzed the root‐associated bacterial community. We found that the rhizosphere communities of the *Fusarium*‐suppressed seedlings were significantly different from the rhizosphere communities of the *Fusarium*‐infected seedlings, while the root endosphere communities were not (**Figure** **5**a). This result once again suggested that *Fusarium* suppression was attributed mainly to the rhizosphere microbes, which was consistent with the results in Figure S3, Supporting Information. Only nine ASVs from the 11‐strain SynM were detected in all of the samples from *Fusarium*‐suppressed seedlings, in which one species (*Chr*) was colonized in significantly lower quantities in the *Fusarium*‐suppressed seedlings than in the *Fusarium*‐infected seedlings (Figure 5b). We then elucidated the *Fusarium* inhibition of these nine species (ASVs) on plates. The inhibition zone assay revealed that *Bac*, as well as *Bur*, strongly inhibited *Fusarium* growth (Figure S5c, Supporting Information). A facilitated relationship test showed that all the strains except *Chr* significantly promoted the growth of the main antagonistic strain *Bac* (Figure 5c). According to these results, our 11‐strain SynM was optimized as an 8‐strain OptSynM containing *Pseudomonas stutzeri* (*Pse*), *Bacillus amyloliquefaciens* (*Bac*), *Lysobacter soli* (*Lys*), *Burkholderia cenocepacia* (*Bur*), *Enterobacter ludwigii* (*Ent*), *Pseudoxanthomonas japonensis* (*Pth*), *Stenotrophomonas maltophilia* (*Ste*), and *Acinetobacter baumannii* (*Aci*).

Inoculation of each individual strain of the OptSynM also revealed that *Bac* and *Bur* could strongly inhibit *Fusarium* in planta, but all of them were less effective for seed‐borne *Fusarium* suppression than the OptSynM (Figure 5d). Moreover, we found that the concentrations of jasmonic acid (JA) and salicylic acid (SA) were not significantly different between germ‐free and strain‐colonized seedling leaves (Figure S6a, Supporting Information), suggesting that OptSynM inoculation or not did not significantly changed the two main defense signaling pathways in plant when facing the endophytic *Fusarium*. Interestingly, inoculation of the “one strain‐knockout” OptSynMs resulted in decreased inhibition rates, in which the “*Bac*‐knockout” OptSynM (−*Bac*, OptSynM without *Bac*) showed the lowest inhibition rate, even lower than inoculation with *Bac* alone (Figure 5d). These results suggested that this OptSynM was a simplified microbiota, in which any member loss will decrease the inhibitory effect, and the antagonism from *Bac* was demonstrated to be the main biological control effect on suppressing the seed‐borne *Fusarium* among this OptSynM. We further tested the general inhibitory function of this OptSynM on the seed‐borne *Fusarium* in all collected maize cultivars and verified that the seed‐borne *Fusarium* could be well suppressed in all cultivars (Figure S6b, Supporting Information). In addition, the inhibitory function of this OptSynM was also active under natural soil conditions (Figure S6c, Supporting Information).

### 2.4 Cooperative Pattern of the OptSynM

**Figure 6:**
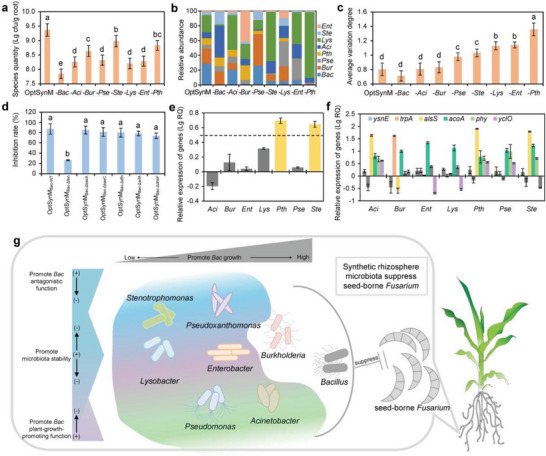
Cooperative pattern of the synthetic microbiota. a) The total colonization quantity, b) the proportion based composition, and c) the compositional average variation degree of the OptSynM and “one strain‐knockout” OptSynMs in the rhizosphere. OptSynM, the 8‐strain OptSynM. −*Bac*, −*Aci*, −*Ent*, −*Pth*, −*Bur*, −*Pse*, −*Lys*, and −*Ste* represent the “one strain‐knockout” OptSynMs obtained by removing each indicated strain from the 8‐strain OptSynM. d) The seed‐borne *Fusarium* inhibition rate of the “mutant‐replaced” OptSynMs. OptSynM*_Bac_*_‐WT_ represents the 8‐strain OptSynM. OptSynM*_Bac‐Δfen_*, OptSynM*_Bac‐ΔbacA_*, OptSynM*_Bac‐Δdfn_*, OptSynM*_Bac‐ΔdhbF_*, OptSynM*_Bac‐ΔbaeC_*, and OptSynM*_Bac‐ΔsrfA_* represent the 8‐strain microbiota, in which *Bac* is replaced by the mutants of *Bac‐Δfen*, *Bac‐ΔbacA*, *Bac‐Δdfn*, *Bac‐ΔdhbF*, *Bac‐ΔbaeC*, and *Bac‐ΔsrfA*, respectively. e) The transcriptional profiles of the antagonistic related *fen* gene from the *Bac* strain under the induction of the supernatants of seven other strains in OptSynM. f) The transcriptional profiles of six plant‐growth‐promoting related genes in the *Bac* strain under the induction of the supernatants of seven other strains in OptSynM. An absolute value of the relative expression of one gene (|LgRQ|) less than 0.5 was considered not remarkably up‐ or down‐regulated (grey pillar). The detailed information on the antagonistic and plant‐growth‐promoting related genes is listed in Table S6, Supporting Information. Different letters above bars indicate significant differences (*p* < 0.05) according to Duncan's multiple comparison. Error bars represent standard deviations (*n* = 4, 4, 3, 6 and 6 in [a], [c], [d], [e], and [f], respectively). g) A conceptual model of the cooperative pattern of the 8‐strain synthetic microbiota. This synthetic microbiota contains a key strain harboring antagonistic and plant‐growth‐promoting functions (*Bac*) and members that promote growth (*Bur* among others), antagonistic functions (*Pth* and *Ste*), and plant‐growth‐promoting functions (*Aci*, *Pth*, *Pse*, and *Ste*) of the key *Bac* strain, as well as members that promote the stability of the synthetic microbiota (*Pth*, *Ent*, and *Lys*). This conceptual model shows how this synthetic microbiota is organized to be stable and efficient in suppressing seed‐borne *Fusarium* and promoting maize growth.

To evaluate the stability of the OptSynM, we further quantified the in planta colonization of the strains in OptSynM and its “one strain‐knockout” OptSynMs by real‐time quantitative polymerase chain reaction (RT‐qPCR) (Table S5, Supporting Information). We found that the OptSynM colonization quantity was decreased after strain knockout (**Figure** **6**a and Figure S7a, Supporting Information). In addition, the proportional composition varied after “strain‐knockout” (Figure 6b). The average variation degree (AVD, see details in the Experimental Section) indicated that *Pth*, *Ent*, and *Lys* knockout significantly increased compositional variations in the OptSynM (Figure 6c), suggesting the importance of these strains in maintaining the stability of OptSynM.

Genome analysis demonstrated that this *Bac* species contains a set of antagonistic and plant‐growth‐promoting related genes (Table S6, Supporting Information). To confirm which antagonistic gene in *Bac* dominates seed‐borne *Fusarium* suppression, mutants of every antagonistic gene were constructed. Further inhibition zone assays of the mutants (Figure S7b, Supporting Information) and gene‐encoded products (Figure S7c, Supporting Information) against *Fusarium* on plates and the inhibition rate assessment by inoculation with the “mutant‐replaced” OptSynM (Figure 6d) revealed that *fen* gene‐encoded fengycin synthesis dominated *Fusarium* suppression.

We then investigated whether the antagonistic and plant‐growth‐promoting functions of *Bac* were affected by the other strains in OptSynM. In vitro culture‐based transcriptional expression of the antagonistic related *fen* gene and six plant‐growth‐promoting related genes in *Bac* (Table S6, Supporting Information) was evaluated under the induction of supernatants from the other seven strains. We found that *Pth* and *Ste* promoted the antagonistic (Figure 6e) and that *Aci*, *Pth*, *Pse*, and *Ste* promoted the plant‐growth‐promoting functions of *Bac* (Figure 6f). We then established strain combinations of *Pth*+*Ste* (antagonistic promotion), *Aci*+*Pse* (plant growth promotion), and *Pth*+*Ste*+*Aci*+*Pse* (antagonistic and plant growth promotion) based on their functional intensification roles on *Bac* (Table S7, Supporting Information) and confirmed their contributions to the functional performance of *Bac* in planta (Figure S7d, Supporting Information).

Taken together, our SynM was simplified and well organized and designed to be stable and efficient in seed‐borne *Fusarium* suppression, in which *Bac* is the key functional bacterium, accomplished by including members that distinctively promote the stability (*Pth*, *Ent*, and *Lys*) and members that inherently promote the growth (*Bur*) and functional gene expression (*Pth* and *Ste* promote the antagonistic functions, and *Aci*, *Pth*, *Pse*, and *Ste* promote the plant‐growth‐promoting functions) of the key bacterium (Figure 6g).

## 3 Discussion

The mutualistic and symbiotic relationships between the microbiota and host plant are essential for plant health.^[^ ^14^, ^25^, ^26^ ^]^ There is a need to properly understand the compositional and functional characteristics of the cropping soil microbiome to manipulate root‐microbe interactions to suppress soil‐ and seed‐borne crop diseases.^[^ ^27^ ^]^ These interactions can be studied by inoculating plants with SynMs in axenic systems, which is a practical method to validate observations of root microbiome patterns without considering the myriad of complex microbial interactions in natural soils.^[^ ^28^ ^]^ The SynM constructed in this study showed consistent performance in suppressing the seed‐borne *Fusarium* in maize. Our study has advantages regarding the innovative approaches for constructing a simplified natural SynM and the unprecedented cooperative pattern to perform a specific function.

The dilution‐to‐extinction approach is an effective way to identify the key functional members of a specific function.^[^ ^29^ ^]^ We acknowledge that the dilution method strongly affects not only the bacterial community but also other organisms, such as fungi and protists, consequently altering biological interactions and ecosystem functions.^[^ ^30^, ^31^ ^]^ Bacterial isolates are the most commonly considered in microbiota construction because they can be easily isolated and cultured. Synthetic microbiotas are generally constructed by pooling in situ isolated bacterial strains that are identified to be positively correlated with the expected functional traits, such as phosphate starvation responses^[^ ^21^ ^]^ and nitrogen use efficiency.^[^ ^22^ ^]^ However, it is rarely considered to construct a SynM with simplified and stable features.^[^ ^24^, ^32^, ^33^ ^]^ Although a SynM comprised of identified taxa may perform the designed plant beneficial functions,^[^ ^22^ ^]^ not all members can colonize the rhizosphere in a robust manner^[^ ^28^ ^]^ and dispensable or redundant members without playing significant roles may also be included.^[^ ^34^ ^]^ Notably, our SynM was constructed through a reductionist approach that focused on a set of core rhizosphere bacteria associated with *Fusarium* suppression. First, a two‐pronged approach was developed to identify the core rhizosphere bacteria. Then, the initial SynM was constructed using the isolates of the core microbes. Finally, the SynM was optimized (OptSynM) to remove the peripheral members through stable coexistence and functional cooperation. The SynM constructed by this strategy exhibits greater advantages of simplification and stability than the irrationally designed microbiota. These experimental approaches may improve our understanding of the modularity of desirable traits within SynMs and consequently improve the possibility in the manufacturing and application of this composite biological agent in agricultural production.^[^ ^35^ ^]^

Previous studies have focused mainly on the overall functions of SynMs.^[^ ^21^, ^22^ ^]^ However, the desired function of a SynM could primarily be attributed to a single microbe^[^ ^18^ ^]^ or an additive effect that involves all members.^[^ ^36^ ^]^ It has been reported that a core consortium of five native bacterial isolates can protect its host against pathogens via complementary traits of producing antifungal compound surfactin and siderophores by different members.^[^ ^37^, ^38^ ^]^ Here, our stable minimal SynM presents a distinctive internal cooperative pattern to efficiently suppress the maize seed‐borne *Fusarium* in planta. *Bac* is an indispensable key strain that performs the desired function, in which *fen* gene‐controlled fengycin synthesis dominates *Fusarium* suppression.^[^ ^39^ ^]^ Fengycin is reported as a lipopeptide and has strong antifungal activity, specifically against filamentous fungi, by damaging the fungal cell membrane.^[^ ^40^, ^41^ ^]^ In particular, the function of the key strain was accomplished by other members that can inherently promote the growth and functional gene expression and can distinctively promote the stability of this SynM under both in vitro and in planta conditions (Figure 6g). *Bacillus* species have been proven to have great advantages in inhibiting various plant pathogens,^[^ ^42^ ^]^ and this inhibitory effect may be enhanced by synergistically interacting with other beneficial rhizospheric microorganisms via cross‐feeding.^[^ ^43^ ^]^ Although these strains in our SynM are isolated from the rhizosphere of healthy seedlings, two of them (*Bur* and *Ste*) are recognized as opportunistic pathogens, which may restrict their potential application in agriculture.^[^ ^44^ ^]^ As a workaround, substitution of the pathogenic strain with nonpathogenic isolate that harbors similar ecofunctions may retain a high efficiency of the desired function; since we found the microbiotas of SynM1 and SynM2 (they were constructed using different ASVs from the same genera as the SynM) also achieved high inhibition rates (Figure S5b, Supporting Information), although the inhibition rates were relatively lower than that of SynM. Notably, the in planta assessment of seed‐borne *Fusarium* suppression by the key strain and SynM suggested that the cooperative pattern was conducive to achieving stable and efficient functions. Clarifying the mechanisms underlying both the stability and efficacy of the SynM will improve its sustainable application in agricultural systems. However, the molecular mechanisms of the bacterial interactions within this microbiota are likely very complex and deserve future study.

## 4 Conclusions

We used a two‐pronged innovative approach to identify the keystone bacteria from the microbiome related to the seed‐borne *Fusarium* suppression function. Then, we isolated these keystone bacterial strains and used the “reductionist” and “strain knock‐out” strategies to simplify the microbiota. We demonstrated that our SynM is simplified and well organized and designed to be stable and efficient in *Fusarium* suppression across all tested maize varieties. Thus, this simplified microbiota could serve as a model bacterial assemblage to study maize root‐microbiota interactions in seed‐borne *Fusarium* suppression. Overall, this study provides an understanding of the assembly of a simplified and stable SynM that performs the desired function and hence will provide a way forward to engineer simple and stable microbial consortia with predictable behavior and robust outcomes.

## 5 Experimental Section

### Soil Collection

Soil samples were collected from Taizhou (120°20′ E, 32°44′ N) in the Jiangsu Province of east China in August 2016. This soil was classified as Orthic Acrisol according to the Food and Agriculture Organization (FAO)/United Nations Educational, Scientific and Cultural Organization (Unesco) System of Soil Classification. Fresh soil was collected from the upper 20 cm (the litter layer was removed) of the field. All samples were sieved through a 2 mm sieve to remove visible plant tissues and stones. The soils were then placed on ice in a portable storage box for immediate transport to the laboratory. Soil subsamples for the measurement of physicochemical properties and for use in pot experiments were air‐dried and stored at room temperature (25 °C). The soil had a pH of 7.53 (soil‐to‐water ratio of 1:5) and contained 1.59% organic matter (potassium dichromate volumetric method), 77.6 mg kg^−1^ N (alkaline hydrolyzable diffusion method), 12.5 mg kg^−1^ P (extracted with sodium bicarbonate and determined using the molybdenum blue method), and 252.5 mg kg^−1^ K (extracted with ammonium acetate and determined using flame photometry). The sterile soil was prepared by *γ*‐irradiation (>50 kGray) (Xiyue Radiation Technology Co., Ltd., Nanjing, China).^[^ ^45^ ^]^

### Maize Seed Surface Sterilization and Germination

Before surface sterilization, the maize seeds were soaked in sterile water (25 °C) for 6 h. For surface sterilization, the seeds were soaked in 70% ethanol for 1 min and rinsed extensively three times in sterile distilled water. Then, the seeds were soaked in 2% sodium hypochlorite solution for 10 min and rinsed extensively three times in sterile distilled water. After surface sterilization, all seeds were placed onto a sterile plate filled with 5 mL of sterile water for germination and incubated in an artificial climate chamber under 16 h light at 22 °C and 8 h dark at 18 °C for 4 days.

### Cultivation Microcosm Setup and Maize Growth

The cultivation microcosm was established using a 1 L transparent bottle filled with 500 g soil (sterile, non‐sterile, or inoculated non‐sterile soil) (Figure 1b). For the inoculated microcosms, all microcosms were incubated after inoculation at 20 °C and at 45% field capacity in the dark for 45 days for microbial community recovery before the 4‐day‐old seedlings were transplanted. After germination, a 4‐day‐old seedling was transplanted into one microcosm. During the entire incubation period, the bottles were covered by a semipermeable membrane for air exchange. Bottles were only opened for experimental manipulation (such as bacterial inoculation and watering) in a biological hood with the semipermeable membrane replaced after each opening. All bottles were spatially randomized and placed in an artificial climate chamber under 16 h light at 22 °C and 8 h dark at 18 °C. Sterile distilled water was added to simulate rain water to maintain the moisture level at 45% field capacity during the cultivation period.^[^ ^46^ ^]^ Three microcosms containing sterile soil without transplanting plants (no inoculation but watering regularly) served as a negative control to confirm that there was no environmental microbial contamination during the cultivation period through the weekly sterility test on agar plates.

### Plant Physiological Characterization

Plant height, shoot fresh weight, and the activity of antioxidant enzymes were detected using 12‐day‐old maize seedlings. To detect the enzyme activity, the fresh shoots were homogenized with extraction buffer (50 mm phosphate buffer containing 1 mm ascorbic acid and 1 mm ethylenediaminetetraacetic acid) and centrifuged at 15 000 g for 15 min at 4 °C. The supernatant was then used to detect the enzymatic activity. A total of three antioxidant enzymatic activities of POD (EC 1.11.1.7), SOD (EC 1.15.1.1), and CAT (EC 1.11.1.6) were detected using enzyme‐specific commercial reagent kits (Suzhou Comin Biotechnology Co., Ltd., Suzhou, China).

### Bulk Soil, Rhizosphere, and Root Endosphere Sampling

Bulk soil samples were collected from the control pots at 2 cm below the soil surface. Each sample was preserved in a clean and sterile 2‐mL centrifuge tube.

For rhizosphere sampling, the bulk soil was manually removed, leaving ≈1 mm of soil on the roots. The roots were placed in a clean and sterile 50‐mL tube containing 30 mL of PBS‐S solution and then vortexed (Vortex‐Genie 2, Scientific Industries, Bohemia, NY, USA) for 15 s at maximum speed. Next, the plant tissues and large sediments in the rhizosphere soil were separated using a new clean and sterile 50‐mL tube with a 100 µm nylon mesh cell strainer,^[^ ^47^ ^]^ followed by a repeated step using 10 mL of PBS‐S solution. The turbid filtrate was then centrifuged for 15 min at 3500 × *g* to pellet the rhizosphere sediment and microbes. The pellet was resuspended in 1 mL of PBS‐S solution, transferred to a 2‐mL microfuge tube, and centrifuged for 5 min at 10 000 × g to form a firm pellet.

The root endosphere sample was collected as clean root tissue without the rhizoplane microbes. The root tissues collected after cell strainer filtering were transferred to a new sterile 50‐mL tube containing 30 mL of PBS‐S and sonicated at 60 Hz for 3 min (30 s sonication and 30 s break, three cycles) to remove the rhizoplane microbes. The roots were then transferred to a 2‐mL centrifuge tube and flash‐frozen in liquid nitrogen.

The soil, rhizosphere, and root endosphere microbial communities were detected by amplicon sequencing using the DNA extracted from the bulk soil, rhizosphere, and root endosphere samples, respectively. All samples were then flash‐frozen in liquid nitrogen and stored at −80 °C until DNA extraction.

### DNA Extraction

The frozen root endosphere samples were preprocessed using a plant grinder (DHS TL 2020, 0401261, DHS Technology Co., Ltd., Beijing, China) at 1800 rpm for 5 min with 30 s vibration and 30 s break in five cycles. Total DNA of the soil, rhizosphere, and root endosphere samples was extracted from 0.25 g of bulk soil, rhizosphere soil, and root endosphere samples, respectively, using the QIAGEN DNeasy PowerSoil Kit (Ref: 12888‐100, Hilden, Germany). To minimize the DNA extraction bias, three successive DNA extractions of each sample were pooled. The DNA quality was assessed according to the 260/280 and 260/230‐nm absorbance ratios by a NanoDrop ND‐2000 spectrophotometer (NanoDrop, ND2000, Thermo Scientific, Wilmington, DE, USA).

### Amplicon Sequencing and Data Processing

The composition of root‐associated bacterial and fungal communities was detected using ribosomal RNA gene amplicon sequencing. The primers for the V3‐V4 hypervariable region of the bacterial 16S rRNA gene (338F: 5′‐ACTCCTACGGGAGGCAGCA‐3′ and 806R: 5′‐GGACTACHVGGGTWTCTAAT‐3′) were used to assess the composition and diversity of the bacterial community, and the primers for the fungal ITS2 region (ITS3: 5′‐GCATCGATGAAGAACGCAGC‐3′ and ITS4: 5′‐TCCTCCGCTTATTGATATGC‐3′) were used to assess the composition and diversity of the fungal community on an Illumina MiSeq instrument (300‐bp paired‐end reads).

The raw sequencing data were processed using the UPARSE pipeline (http://drive5.com/usearch/manual/uparse_pipeline.html)^[^ ^48^ ^]^ with assembled paired reads, quality control (QC), trim length, dereplication, and the removal of singletons and chimeric sequences. The remaining sequences were used to extract ASVs from each sample by the open‐source software package DADA2 (v.1.8).^[^ ^49^ ^]^ The taxonomic assignment for each ASV was performed using the Ribosomal Database Project (RDP) database (RDPClassifier_16S_trainsetNo16, Release date: 2016‐07‐12) (https://sourceforge.net/projects/rdp‐classifier/) for the bacterial community and the UNITE database (Release date: 2017‐6‐28) (http://unite.ut.ee/) for the fungal community. The DNA sequences were deposited in the National Center for Biotechnology Information (NCBI) under accession numbers PRJNA664660 and PRJNA664749.

The ASV table was rarefied at 10 000 reads per sample. The relative abundance of a phylogenetic group (or ASV) was defined as the number of sequences affiliated with that group (or ASV) divided by the total number of sequences per sample. The *α*‐diversity (Shannon diversity and richness index) calculation, Bray–Curtis distance‐based principal coordinate analysis (PCoA) analysis, and permutational multivariate analysis of variance of the bacterial and fungal communities were performed using the rarefied ASV table by the “vegan” R package (v.2.5‐2) (https://cran.r‐project.org/package=vegan). FastUnifrac^[^ ^50^ ^]^ was used to calculate the phylogenetic community dissimilarity (*β*‐diversity).

### Inhibition Rate Calculation

A maize seedling was classified as a *Fusarium*‐infected seedling when the *Fusarium* relative abundance was above 80% in the root‐associated community and as a *Fusarium*‐suppressed seedling when it was below 20%. The seedlings with rhizospheric *Fusarium* relative abundances between 20% and 80% were discarded. The *Fusarium*‐infected rhizosphere and root endosphere samples were collected from *Fusarium*‐infected seedlings and the *Fusarium*‐suppressed rhizosphere and root endosphere samples were collected from *Fusarium*‐suppressed seedlings (see details in the Bulk soil, Rhizosphere, and Root Endosphere Sampling section). The relative abundance of *Fusarium* in a community was determined by amplicon sequencing of the fungal ITS2 region (see details in the Amplicon Sequencing and Data Processing section). The inhibition rate was calculated by dividing the number of *Fusarium*‐suppressed seedlings by the total number of seedlings.

### Experimental Procedure: Identify Seed‐Borne *Fusarium*

The experimental procedure of this study is shown in Figure 1a.

*Isolation of seed‐borne fungi*: A total of 20 randomly selected seeds from four batches (five seeds from each batch) of each cultivar were used for isolation of seed‐borne fungi (Table S1, Supporting Information). Three fungal strains were isolated from each seed; hence, a total of 60 strains for each cultivar were isolated. The surface‐sterilized maize seeds were germinated and cultivated in MS medium, and the Petri dishes were incubated in darkness at 28 °C. The germinated seeds were covered by visible fungal mycelia in a few days, and the emerged hyphal tips were then carefully picked and further subcultured on potato‐dextrose agar. These isolates were subcultured twice for strain purification before species identification. Fungal DNA was extracted using a Fungal DNA Kit (OMEGA D3390‐01, Shanghai, China) and the species were identified using ITS sequences (ITS1: 5′‐TCCGTAGGTGAACCTGCGG‐3′ and ITS4: 5′‐TCCTCCGCTTATTGATATGC‐3′).^[^ ^51^ ^]^ The sequence alignment for seed‐borne *Fusarium* identification was carried out with the ClustalX program based on the neighbor‐joining method. The unrooted phylogenetic trees were created using the MEGA6.0 program with 1000 bootstrap replications. The ITS sequence data indicated that these fungal strains were the same within the same cultivar (although from different batches), and all strains were identified as *Fusarium* spp. The cultures of the pure fungal strains were stored as a suspension of mycelium and spores in 50% v/v glycerol at −70 °C.

*Time‐series experiment setup*: The 4‐day‐old seedlings (Fengtian843; FT) were transplanted into microcosms containing sterile or non‐sterile soil. 20 replicated pots were established for sterile and non‐sterile soil cultivation, with four randomly selected pots for sampling at each sampling period. The rhizosphere and root endosphere samples were collected at successive intervals (0, 6, 9, 12, 16, and 20 days), and the root endosphere sample at Day 0 was recognized as the equivalent of seeds.^[^ ^46^ ^]^ The bulk soil samples were collected before (Day 0) and after (Day 20) the cultivation period.

### Experimental Procedure: Identify Core Rhizobacteria

*Progressive dilution experiment*: A 10^−1^ soil suspension was made by mixing 20 g fresh soil with 180 mL sterile distilled water. This 10^−1^ suspension was then serially diluted to create progressively diluted 10^−2^, 10^−2^, 10^−3^, 10^−4^, and 10^−5^ suspensions,^[^ ^29^ ^]^ which were then inoculated into the microcosms containing sterile soil. Two sets of microcosm repetitions were established for each dilution level. Each set of microcosm repetition contained 50 microcosms (seedlings). Another 100 microcosms containing non‐sterile soil (Dil0) served as the undiluted control. Therefore, 600 microcosms were established in the progressive dilution experiment. After inoculating the soil suspension, all microcosms were incubated at 20 °C and at 45% field capacity in the dark for 45 days for microbial community recovery before the 4‐day‐old seedlings were transplanted. Two replicated rhizosphere and root endosphere samples at each dilution level were collected at Day 12 after transplanting and each sample was pooled, consisting of five randomly selected seedlings.

*PLS‐DA and RF machine learning analysis*: The best discriminant performance of individual rhizosphere bacterial taxa across seed‐borne *Fusarium* infection and *Fusarium* suppression seedlings was acquired using PLS‐DA^[^ ^52^ ^]^ and random forest (RF)^[^ ^53^ ^]^ methods. The relative abundance of rhizosphere bacterial taxa was used as independent variables to generate the classification model for the infected or suppressed state. The PLS‐DA and RF were performed using SIMCA‐P software (v.11.5) and the rfcv() and varImpPlot() functions in the “randomForest” (mtry = 15, ntree = 800) R package (v.4.6‐14), respectively.

*Root exudate collection and compositional analysis*: A root splitting device separated by a partition with one half of the root growing in the soil (sterile or non‐sterile soil) and the other half growing in sterile distilled water (Figure 4a) was applied to simulate the soil in situ cultivation and minimize the influence of root‐washing. All seedlings were placed in an artificial climate chamber under 16 h light at 22 °C and 8 h dark at 18 °C. Three replicated root exudate samples were collected from the sterile distilled water side at the one‐leaf (V1), two‐leaf (V2), and three‐leaf (V3) growth stages. The collected samples were filtered through a 0.45‐µm membrane (Millipore, Burlington, MA, USA). A total of 100 µL of each filtered sample was plated on Luria Bertasni (LB) agar plates (incubated at 30 °C for 24 h) to test sterility. Root exudate samples were then lyophilized and stored at −80 °C.

Root exudate samples were analyzed using gas chromatography time‐of‐flight mass spectrometry (GC‐TOF‐MS). Each lyophilized sample was transferred into a 2‐mL tube and mixed with 1000 µL of a precooled extraction mixture (methanol/distilled (d)H_2_O [v:v] = 3:1) and 10 µL of internal standard (adonitol, 0.5 mg mL^−1^). The samples were then vortexed for 30 s and homogenized for 4 min at 35 Hz in a ball mill, followed by ultrasonication for 5 min in ice‐water. After centrifugation at 4 °C for 15 min at 11 000 rpm, the supernatants were transferred to new sterile tubes. A total of 150 µL supernatant from each sample was extracted and mixed as a QC sample for a total of three QC samples. All samples were evaporated in a vacuum concentrator, and 60 µL of methoxyethylmethylamine hydrochloride (20 mg mL^−1^ in pyridine) was added. The samples were then incubated at 80 °C for 30 min and derivatized by 80 µL of *N*,*O*‐bis(trimethylsilyl)acetamide (BSTFA) reagent (1% trimethylchlorosilane v/v) at 70 °C for 1.5 h. After cooling to room temperature, 10 µL of FAMEs (in chloroform) was added to the samples and then analyzed by GC‐TOF‐MS. The GC‐TOF‐MS analysis was performed using an Agilent 7890 gas chromatograph mass spectrometer with a DB‐5MS capillary column (Agilent, Santa Clara, CA, USA). A total of 1 µL of sample was injected in splitless mode using helium as the carrier gas. The front inlet purge flow was 3 mL min^−1^ and the gas flow rate was 1 mL min^−1^ through the column. The initial temperature was 50 °C for 1 min, then raised to 310 °C at a rate of 10 °C min^−1^ and kept at 310 °C for 8 min. The front injection, transfer line, and ion source temperatures were 280, 280, and 250 °C, respectively. The electron energy was −70 eV, and the mass spectrometry data were collected in full‐scan mode with an *m*/*z* range of 50–500 at a rate of 12.5 spectra per second after a solvent delay of 6.25 min.

The raw data were analyzed through peak extraction, baseline adjustment, deconvolution, alignment, and integration using Chroma TOF software (V 4.3x, LECO, St. Joseph, MI, USA), and the metabolite was identified by matching the mass spectrum and retention index using the LECO‐Fiehn Rtx5 database. The peaks detected in less than half of the QC samples or relative standard deviation >30% in the QC samples were removed.^[^ ^54^ ^]^ The remaining data were then analyzed by STAMP software (V2.1.3, June 26, 2015) to elucidate which root exudates between the *Fusarium*‐suppressed and *Fusarium*‐infected seedlings were significantly different.

*Soil incubation and chemotaxis assay using typical compounds of root exudate*: The typical compounds of maize root exudates (Table S3, Supporting Information), which were significantly changed (*p* < 0.001) and in high proportions, were selected to conduct the soil incubation and chemotaxis assay. For the soil incubation, watery solutions of the mixtures were prepared containing each of the selected compounds in an equal final dosage of 1.0 mm. Four treatments (typical compound mixtures of T_InfectedEx_V2, T_InfectedEx_All, T_SuppressedEx_V2, and T_SuppressedEx_All) and a control (sterile distilled water) were applied in eight repetitions. 10 g of fresh soil was placed into each well of a 6‐well plate. The plates were preincubated in a growth chamber at 30 °C for 1 week, and each well then received 0.5 mL of exudate compound solution once every 3 days and was incubated in a growth chamber at 30 °C for another 45 days. Soil samples were collected from the wells at the end of the incubation period.

For the chemotaxis assay, capillary tubes containing the compound mixtures used in the soil incubation assay were pushed into a suspension of bacteria.^[^ ^55^ ^]^ The bacterial suspension was prepared the same as the bacterial suspension in the progressive dilution experiment. The chemotaxis assay progressed for 40 min in four replicates. Bacterial samples were then collected from the chemotactic pools. All samples from the soil incubation and chemotaxis assays were stored at −80 °C until DNA extraction.

### Experimental Procedure: SynM Construction

*Rhizospheric bacterial isolation*: Rhizosphere samples of five *Fusarium*‐suppressed maize seedlings (randomly selected from the undiluted control [non‐sterile soil] in the progressive dilution experiment) were pooled for rhizospheric bacterial isolation. Samples were resuspended in PBS‐S solution and progressively diluted to 10^−5^ (the most appropriate dilution level in this study). The supernatant of the 10^−5^ suspension was distributed and cultivated in 96‐well microtiter plates in 1:10 v/v tryptic soy broth (TSB) for 20 days at room temperature. A two‐step barcoded PCR protocol in combination with Illumina HiSeq was adopted to define the V3‐V4 sequences of bacterial 16S rRNA genes of the rhizosphere bacteria.^[^ ^22^ ^]^ The sequences of bacterial isolates were compared with those of the rhizosphere bacterial ASVs at a similarity level of 100%. Isolates were purified on the respective solidified media before an individual colony was further utilized for microbiota construction and genome sequencing. The full‐length 16S rRNA gene of the colonies was sequenced to confirm the correctness of the purified strain. The radiation trees of the ASVs that were detected in the rhizosphere of *Fusarium*‐suppressed seedlings and the isolated bacteria were constructed by FastTree (v.2.1.3)^[^ ^56^ ^]^ and visualized by iTol (https://itol.embl.de/).

*Synthetic microbiota (SynM) construction*: A total of 237 isolated strains were used for SynM construction. Except for the seed‐borne *Fusarium* suppression‐associated 11 core ASVs, five control SynMs were also constructed (Table S4, Supporting Information). First, the enrichment or depletion of an ASV was identified through comparative analysis of the relative abundance between the *Fusarium*‐suppressed and *Fusarium*‐infected rhizosphere communities in the progressive dilution experiment. SynM1 and SynM2 were constructed by replacing each of the core ASVs with the ASVs from the same genera. SynM3 was constructed by randomly selected ASVs that were enriched (but were not identified as core ASVs) in *Fusarium*‐suppressed seedlings. SynM4 was constructed by randomly selected ASVs that were depleted in *Fusarium*‐suppressed seedlings. SynM5 was constructed by randomly selected ASVs from all the unique strains.

*Inhibition rate assay of SynM*: For inoculum preparation, each bacterium was grown in TSB broth and then centrifuged at 3000 rpm for 20 min when the OD_600_ was 1.0. The supernatant was discarded and the sediment was resuspended in sterile distilled water to 10^9^ cfu mL^−1^. Then, the inocula of six SynMs were generated by pooling the resuspended 11 strains in equal volumes (Table S4, Supporting Information). For the inhibitory rate assay, the cultivation microcosms were established using sterile soil and the bacterial inocula were inoculated at a final concentration of 10^8^ cfu g^−1^ soil. 60 maize seedlings were planted for each inoculum and repeated the process three times. The inhibition rate was calculated as previously described.

### Experimental Procedure: SynM Simplification

*Robust colonization assay*: The bacterial communities of the rhizosphere and root endosphere samples from the SynM‐inoculated microcosms were sequenced. In the inhibition rate assay of SynM, 24 *Fusarium*‐infected and 153 *Fusarium*‐suppressed seedlings were observed out of 180 seedlings (60 maize seedlings in three repetitions, three seedlings discarded). 12 *Fusarium*‐infected and 16 *Fusarium*‐suppressed samples were sampled (each sample was pooled by two randomly selected seedlings). The successful colonization of a strain was confirmed when the bacterial ASVs were compared with the sequences of the inoculated bacterial isolates at a similarity level of 100%.

*Inhibition zone assay*: The antagonistic activities of the nine robust colonized strains against *Fusarium* were examined by inhibition zone assays on potato dextrose agar (PDA) plates. A plug (0.5 cm in diameter) of *Fusarium* from a 7‐day‐old *Fusarium*‐growing PDA plate was placed in the center. The strains were then inoculated onto equidistant points at the edge of a Petri plate. Four points on each plate were inoculated, and the plate was repeated twice. The antagonistic effect was identified after 7 days.

*Facilitate relationship test*: To obtain the supernatant of the strains, each bacterium was grown in TSB broth and then centrifuged at 7500 rpm for 15 min when the OD_600_ was 1.0. The supernatant was harvested and filtered with sterile 0.22‐µm filters. For growth curve measurement, liquid culture in 96‐well microtiter plates was analyzed in a Bioscreen growth curve analyzer (Oy Growthcurves Ab Ltd., Varsinais‐Suomi, Finland) at 30 °C. Each well contained 198 µL supernatant (equivalent TSB as control) and 2 µL TSB‐resuspended *Bac* strain (OD_600_ = 1.0) in logarithmic growth phase. Ten replicates were performed for each treatment.

*Verify the simplification of OptSynM*: To test the general inhibitory function of the OptSynM on seed‐borne *Fusarium* in all of the collected maize cultivars, the maize seedlings were transplanted into microcosms that were established by inoculating OptSynM into sterile soil. 20 maize seedlings were tested for each cultivar. The microcosms containing sterile soil and non‐sterile soil served as negative and positive controls, respectively.

The inhibition rate assay of “one strain‐knockout” OptSynM and each individual strain of the OptSynM were performed in cultivation microcosms. These “one strain‐knockout” OptSynMs were constructed by removing one of the eight species in OptSynM. Therefore, eight “one strain‐knockout” OptSynMs were constructed, each containing seven strains. The inocula were prepared as previously described at a concentration of 10^9^ cfu mL^−1^. The cultivation microcosms were established using sterile soil and the bacterial inocula were inoculated at a final concentration of 10^8^ cfu g^−1^ soil. 60 maize seedlings were planted for each inoculum and repeated the process three times. The inhibition rate was calculated as previously described.

*JA and SA measurement*: The JA and SA concentrations were measured on a SpectraMax i3x analysis system (Molecular Devices Corporation, San Jose, CA, USA).^[^ ^57^ ^]^ Briefly, the collected plant leaves were flash‐frozen in liquid nitrogen and smashed. The smashed plant tissue was then blended into sodium phosphate buffer (pH 7.0) at a weight/volume ratio of 1:9. The mixture was centrifuged at 3000 × *g* for 20 min before the supernatant was harvested for the detection of SA and JA. To detect the SA and JA concentrations, an enzyme‐linked immunosorbent assay (ELISA) (Lengton Bioscience Co., Ltd., Shanghai, China) was used. The horseradish POD conjugate reagent and 50 µL of supernatant were added to each well of the ELISA kit plate. The plate was then incubated at 37 °C for 60 min and the color reacted for 10 min at 37 °C in darkness. Finally, the absorbance at 450 nm was measured using the SpectraMax i3x analysis system (Molecular Devices Corporation, CA) and the concentration was calculated based on the standard curve. The concentrations were measured with four repetitions.

### Experimental Procedure: OptSynM Cooperative Pattern

*Bacterial strain genome sequencing and preservation*: The genomes of the eight species in OptSynM (*Pse*, *Bac*, *Lys*, *Bur*, *Ent*, *Pth*, *Ste*, and *Aci*) were sequenced and the genome sequences were deposited in the NCBI under accession number PRJNA822872. These pure strains were deposited in the National Culture Collection Center, the China General Microbiological Culture Collection Center (CGMCC), with CGMCC numbers (CGMCC No.) from 21321 to 21328.

*OptSynM colonization quantification*: The specific primer set for each species was designed based on the unique sequences (Table S5, Supporting Information) and the specificity of each primer set was clarified through conventional PCR and gel electrophoresis. The standard curves were generated between the RT‐qPCR Ct value and Lg transformed colony forming units (Cfu). To draw the standard curve, each bacterium was grown in TSB broth and then centrifuged at 7500 rpm for 15 min when the OD_600_ was 1.0. The supernatant was discarded and the deposit was washed three times using PBS‐S solution. Then, the bacterium was resuspended in PBS‐S solution and diluted in the order of magnitude gradient. Finally, the Ct values were detected on an ABI 7500 real‐time PCR system (Applied Biosystems, Waltham, MA, USA), and the Cfu values were detected by the plate count method for each dilution level.

To detect the colonization quantity of the OptSynM in planta, the total DNA of the rhizosphere samples collected from the seedlings in the inhibition rate assay that inoculated the 8‐strain OptSynM and 7‐strain “strain‐knockout” OptSynMs was analyzed. The Ct value of each species was detected with four repetitions using the specific amplification primer sets for each species with ChamQ SYBR qPCR Master Mix (Vazyme Biotech Co., Ltd., Nanjing, China) on an ABI 7500 real‐time PCR system (Applied Biosystems, USA) and the rhizospheric colonization quantity was then calculated according to the standard curve.

*OptSynM stability assay*: The stability of the SynM was calculated based on the relative abundance of each species in the communities. The relative abundance was calculated by dividing the quantity of one species by the quantity of inoculated species. Then, the AVD was calculated using the AVD index,^[^ ^29^ ^]^ which was calculated using the deviation degree of the normally distributed relative abundance of each species after incubation from the initial composition (inoculum).

*Antagonistic activity against Fusarium*: To determine the role of antagonistic substance synthesis genes from *Bac* in the biological control of seed‐borne *Fusarium*, the antagonistic genes were first annotated based on the genome of *Bac* (Table S6, Supporting Information) and the *fen*, *bacA*, *dfn*, *dhbF*, *baeC*, and *srfA* genes were disrupted by double cross‐recombination.^[^ ^39^ ^]^ The antagonistic activities of the *Bac* mutants (*Bac‐Δfen*, *Bac‐ΔbacA*, *Bac‐Δdfn*, *Bac‐ΔdhbF*, *Bac‐ΔbaeC*, and *Bac‐ΔsrfA*) and these gene‐encoded products against *Fusarium* were examined on PDA plates. Each bacterium was grown in TSB broth and then centrifuged at 7500 rpm for 15 min when the OD_600_ was 1.0. The supernatant was harvested and filtered with sterile 0.22‐µm filters to obtain the supernatant of each strain. A 0.1 mL methanol extracted supernatant was dropped into an Oxford cup placed 1 cm from the edge of a Petri plate and allowed to diffuse into agar. A plug (0.5 cm in diameter) of *Fusarium* from a 7‐day‐old *Fusarium*‐growing PDA plate was placed in the center. The plates were then incubated at 25 °C and the antagonistic effect was identified after 7 days.

*Effect of accessory OptSynM members on the gene expression of Bac*: For the transcriptional profile assay in vitro, each bacterium was first grown in TSB broth and then centrifuged at 7500 rpm for 15 min when the OD_600_ was 1.0. The supernatant was harvested and filtered with sterile 0.22‐µm filters. A 10 mL liquid culture of the *Bac* strain (OD_600_ = 1.0) was induced by 200 µL supernatant in a growth chamber at 30 °C and 170 rpm for 40 min. The fermentation broth was then frozen in liquid nitrogen, and the total RNA was extracted using a Bacterial RNA Kit (Omega Bio‐Tek, Norcross, GA, USA). The extracted total RNA was reverse transcribed into cDNA in a 20‐µL reaction volume using a reverse transcription system of HiScript II Q RT SuperMix (Vazyme Biotech Co., Ltd., Nanjing, China) according to the manufacturer's instructions. Transcriptional expression of the antagonistic related *fen* gene and six plant‐growth‐promoting related genes in *Bac* were quantified by RT‐qPCR (Table S6, Supporting Information). RT‐qPCR was performed using an ABI 7500 real‐time PCR system (Applied Biosystems, USA) with ChamQ SYBR qPCR Master Mix (Vazyme Biotech Co., Ltd., Nanjing, China). The 2^−∆∆^ *^Ct^* method was used to analyze the qPCR data, and *recA* was used as the reference gene.^[^ ^58^ ^]^ For the transcriptional profile assay in planta, the total RNA of the rhizosphere microbiota was extracted from rhizosphere samples under the inoculation of different strain combinations (Table S7, Supporting Information) using the QIAGEN RNeasy PowerSoil Kit (Ref: 12866‐25, Germany). Transcriptional expression of the antagonistic related *fen* gene and six plant‐growth‐promoting related genes in *Bac* were quantified by RT‐qPCR using the reverse transcribed cDNA of the rhizospheric total RNA. The relative expression of each gene was analyzed by dividing the gene copy number by the *Bac* colonization quantity.

### Significance Analysis

Duncan's multiple comparisons test was used to calculate significant differences among samples. Two‐sided *t*‐tests were used to calculate the significance of differences between two samples. All statistical analyses were performed using R software (v.4.0.2; https://www.r‐project.org/).

## Conflict of Interest

The authors declare no conflict of interest.

## Author Contributions

W.X. and Y.R. contributed equally to this work. W.X., R.Z., and Q.S. designed the study. Y.R., H.Y., A.M., Z.L., and L.W. performed experimental work and detailed the sampling. Y.R., Z.X., N.Z., Y.M., and H.F. conducted the DNA purification and organized the sequencing. W.X. and Y.R. carried out the bioinformatics and statistical analysis. W.X., Y.R., and R.Z. created the figures and drafted the manuscript. All authors helped review, edit, and complete the manuscript.

## Supporting information

Supporting Information

## Data Availability

The data that support the findings of this study are available in the supplementary material of this article.
