# Supplementary material for: Sustained Inhibition of Maize Seed‐Borne Fusarium Using a Bacillus‐Dominated Rhizospheric Stable Core Microbiota with Unique Cooperative Patterns
Source: Adv Sci (Weinh). 2022 Dec 18;10(5):2205215. doi: 10.1002/advs.202205215 (PMC9929125; doi:10.1002/advs.202205215)
Supplement: Supplementary file 1 — Supporting Information [file ADVS-10-2205215-s001.pdf]

ADVANCED SCIENCE
Open Access

Supporting Information

for *Adv. Sci.*, DOI 10.1002/advs.202205215

Sustained Inhibition of Maize Seed-Borne *Fusarium* Using a *Bacillus*-Dominated Rhizospheric Stable Core Microbiota with Unique Cooperative Patterns

Weibing Xun, Yi Ren, He Yan, Aiyuan Ma, Zihao Liu, Lingling Wang, Nan Zhang, Zhihui Xu, Youzhi Miao, Haichao Feng, Qirong Shen and Ruifu Zhang**

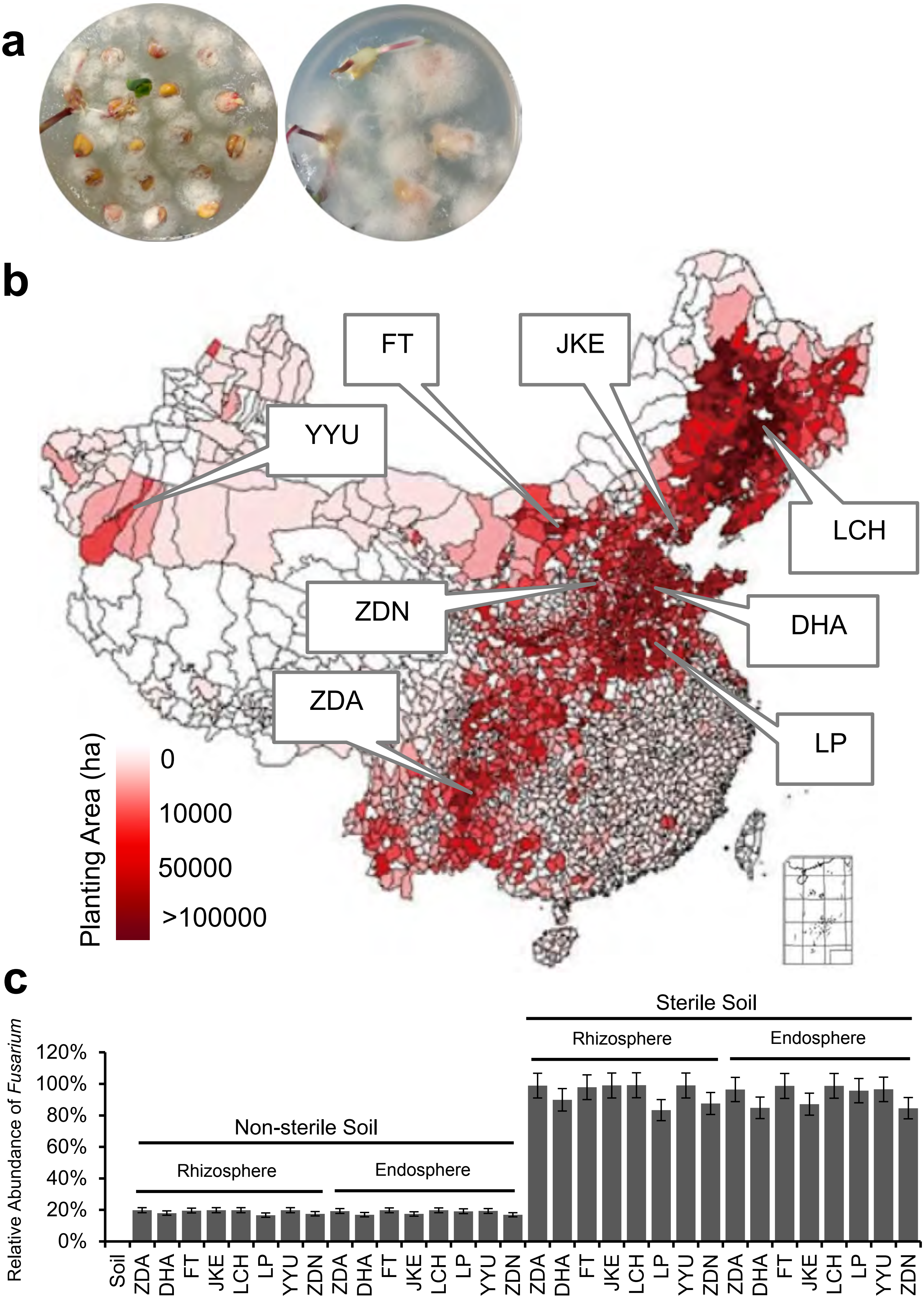

Fig. S1. Seed-borne *Fusarium* in maize seeds across various cultivars. a, The surface-disinfected seeds were covered by fungal mycelium when germinated on MS culture medium. **b**, The planting areas of the eight commercial maize cultivars. **c**, The relative abundance of *Fusarium* in the rhizosphere and root endosphere fungal communities of all maize varieties when cultivated in both non-sterile and sterile soils. The abbreviations of all maize varieties are listed in **Table S1**. Error bars represent standard deviations (n = 6).

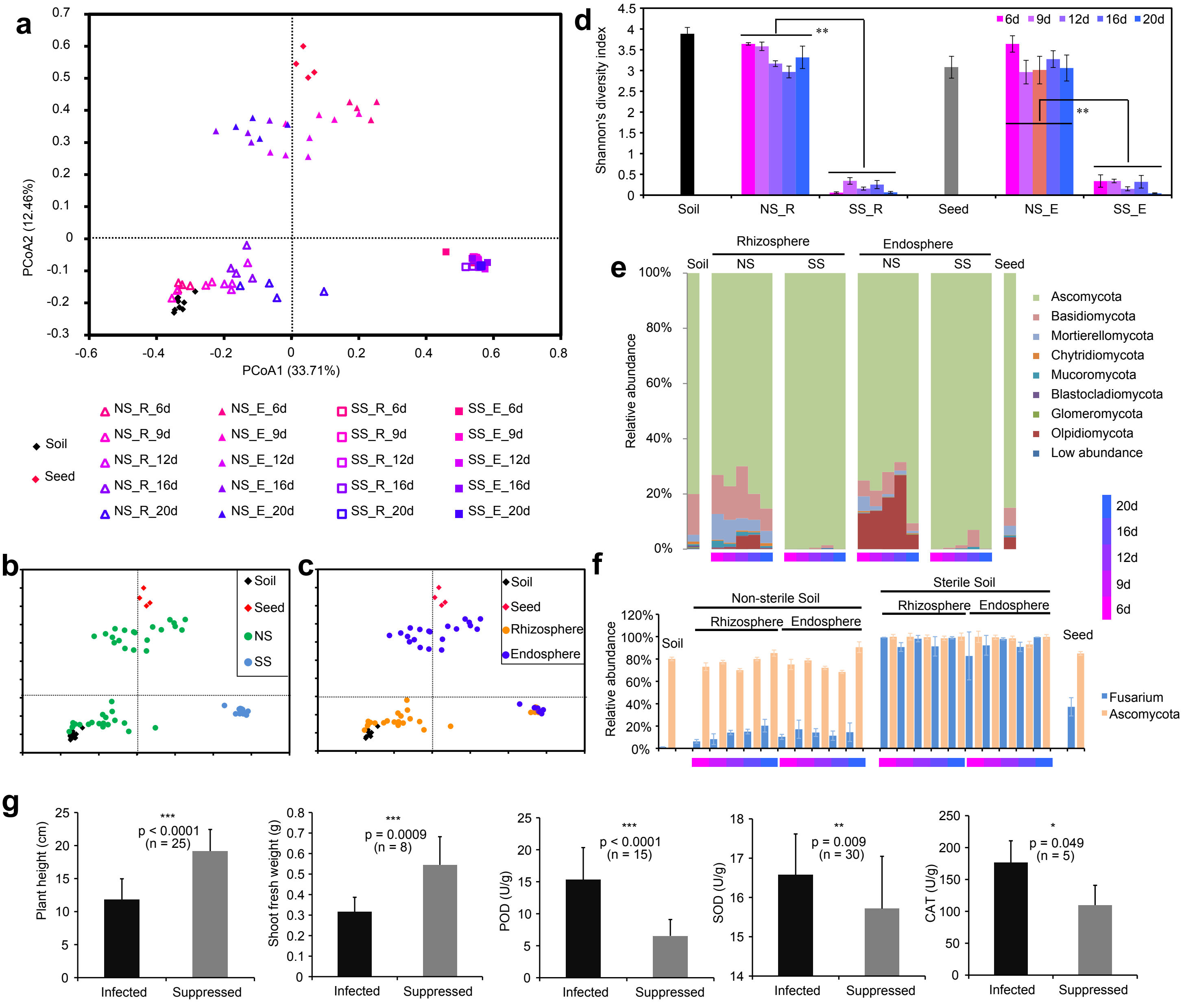

Fig. S2. Root-associated fungal community in sterile and non-sterile soils. a, PCoA of the rhizosphere and root endosphere fungal communities at successive intervals (0, 6, 9, 12, 16, and 20 days) in pot trials. **b**, The same PCoA plot distinguishing the fungal communities in sterile and non-sterile soils. The fungal communities from non-sterile soil were significantly different (ANOSIM R-value = 0.933, p-value < 0.001) from those from sterile soil. In non-sterile soil, the fungal communities were significantly different (ANOSIM R-value = 0.853, p-value < 0.001) between the rhizosphere and root endosphere samples. In sterile soil, the fungal communities were not significantly different (ANOSIM R-value = 0.048, p-value = 0.069) between the rhizosphere and root endosphere samples. **c**, The same PCoA plot distinguishing the rhizosphere and root endosphere fungal communities. NS_R: rhizosphere community in non-sterile soil. SS_R: rhizosphere community in sterile soil. NS_E: root endosphere community in non-sterile soil. SS_E: root endosphere community in sterile soil. **d**, The Shannon diversity of the rhizosphere and root endosphere fungal communities at successive intervals (0, 6, 9, 12, 16, and 20 days) in pot trials. Asterisks indicate significance: **p-value < 0.01 based on two-sided *t*-test. Error bars represent standard deviations (n = 4). **e**, Taxonomic distributions of the bulk soil, seed, and rhizosphere and root endosphere fungal communities at phylum level based on amplicon sequencing data at successive intervals (0, 6, 9, 12, 16, and 20 days) in pot trials. NS: non-sterile soil. SS: sterile soil. **f**, The relative abundance (RA) of the genus *Fusarium* and the phylum Ascomycota in the fungal communities. The phylum Ascomycota was dominant in the bulk soil (RA: 80.0%±1.6%), seed (85.0%±1.7%), non-sterile (68.4%-90.6%) and sterile samples (93.0%-100.0%). The *Fusarium* belonging to the Ascomycota was very low in the bulk soil (1.3%±0.2%) and non-sterile soil (6.3%-20.3%), but relatively higher in the seed (37.2%±8.2%) and the highest in the sterile soil samples (82.8%-99.5%). Error bars represent standard deviations (n = 4). **g**, The plant heights, shoot fresh weights and the enzymatic activity of peroxidase (POD), superoxide dismutase (SOD) and catalase (CAT) in *Fusarium*-infected and *Fusarium*-suppressed seedlings. Asterisks indicate significance: *p-value < 0.05, **p-value < 0.01 and ***p-value < 0.001 based on two-sided *t*-test. Error bars represent standard deviations. n = 25, 8, 15, 30 and 5 (from left to right) biological replicates.

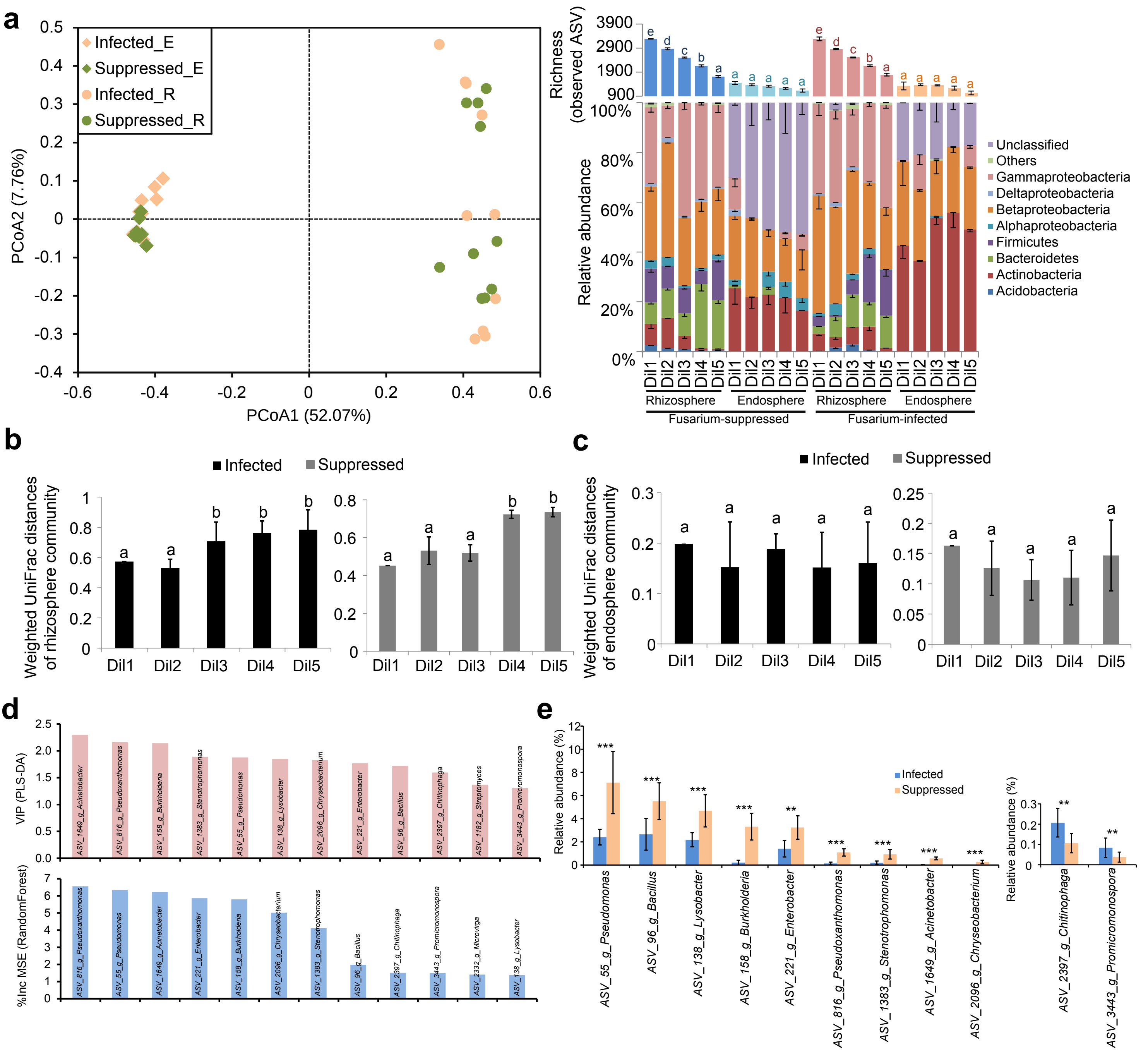

Fig. S3. Root-associated bacterial community in the progressive dilution experiment. **a**, PCoA of the root-associated bacterial communities of the *Fusarium*-infected and *Fusarium*-suppressed groups in the progressive dilution experiment (left). The rhizosphere bacterial communities were significantly different from the root endosphere communities (ANOSIM R-value = 0.997, p-value < 0.001). The communities were not significantly different between the *Fusarium*-infected and *Fusarium*-suppressed groups within both the rhizosphere (ANOSIM R-value = 0.011, p-value = 0.367) and root endosphere (ANOSIM R-value = 0.094, p-value = 0.062) bacterial communities. The bacterial richness (number of observed ASVs) (right-top) and the composition of the root-associated bacterial communities of the *Fusarium*-infected and *Fusarium*-suppressed groups in the progressive dilution experiment (right-bottom). Dil0 indicates the non-sterile soil. Dil1 to Dil5 indicate the dilution level from 10^{-1} to 10^{-5}. **b**, Weighted UniFrac distances between the rhizosphere bacterial communities of 10^{-1} (dilution level) and other dilution levels. **c**, Weighted UniFrac distances between the root endosphere bacterial communities of 10^{-1} (dilution level) and other dilution levels. For instance, Dil1 on the horizontal ordinate indicates the distance between the bacterial communities within 10^{-1}. Dil3 on the horizontal ordinate indicates the distance between the bacterial communities of 10^{-1} and 10^{-3}. Error bars represent standard deviations (n = 2, 4 and 4 in **a**, **b** and **c**, respectively). Different letters above bars indicate significant differences (p-value < 0.05) according to Duncan's multiple comparison. **d**, The top 12 bacterial ASVs were identified by applying partial least squares discrimination analysis (PLS-DA) and random-forest classification (RF) methods of the rhizosphere bacterial communities against *Fusarium*-suppressed and *Fusarium*-infected groups. Biomarker ASVs are ranked in descending order of the accuracy value by the RF method (84.6% accuracy) and PLS-DA (89.3% accuracy) methods. **e**, The relative abundance of the shared indicator taxa enriched in the rhizosphere bacterial communities of the *Fusarium*-suppressed seedlings (left) and the *Fusarium*-infected seedlings (right) by three methods in **a**. Error bars represent standard deviations(n = 10). Asterisks indicate significance: **p-value < 0.01 and ***p-value < 0.001 based on two-sided *t*-test.

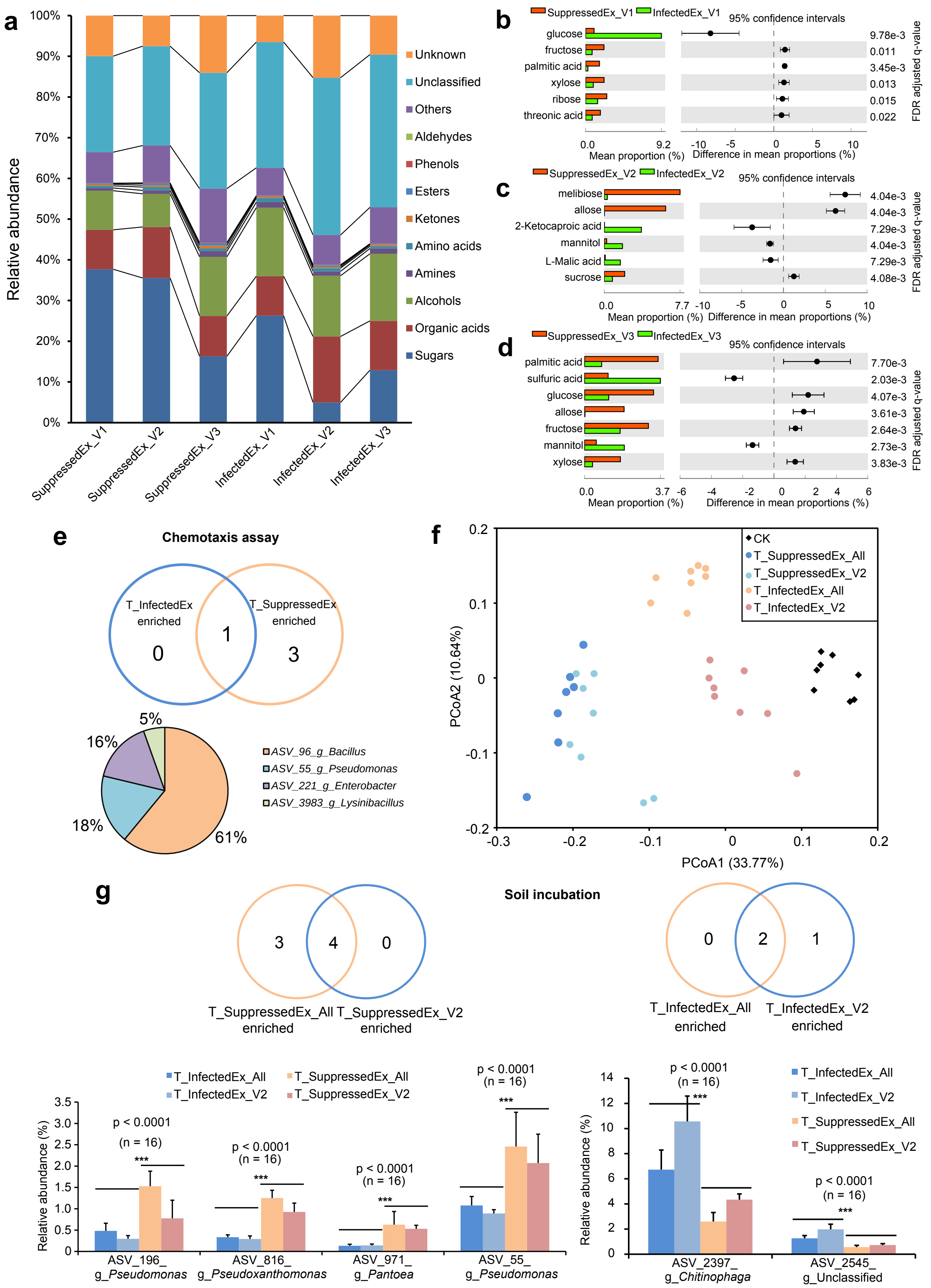

Fig. S4. The composition of root exudate, soil incubation and chemotaxis assay. a, The root exudate composition of *Fusarium*-infected (InfectedEx from sterile soil) and *Fusarium*-suppressed (SuppressedEx from non-sterile soil) seedlings at the one-leaf (V1), two-leaf (V2) and three-leaf (V3) stages. **b**, The typical compounds in high proportions and significantly enriched in SuppressedEx and InfectedEx at the one-leaf (V1) stage. **c**, The typical compounds in high proportions and significantly enriched in SuppressedEx and InfectedEx at the two-leaf (V2) stage. **d**, The typical compounds in high proportions and significantly enriched in SuppressedEx and InfectedEx at the three-leaf (V3) stage. The difference in mean proportions between the groups is shown with 95% confidence intervals (n = 3). **e**, Venn diagram (top) and pie chart (bottom) of ASVs attracted by SuppressedEx and InfectedEx compounds in the chemotaxis assay. The relative abundance of each ASV was determined by amplicon sequencing. **f,** PCoA of the soil bacterial communities. The soils are treated with different mixtures of typical compounds (see details in **Table S3**). CK is the control treated with an equal amount (the same frequency and quantity) of sterile distilled water. The bacterial communities were significantly different after incubation with the typical compounds of SuppressedEx and InfectedEx (ANOSIM R-value = 0.477, p-value < 0.001). **g**, Venn diagram (top) and relative abundance of abundant ASVs (RA > 0.5%) (bottom) that were simultaneously enriched by SuppressedEx (T_SuppressedEx_All and T_SuppressedEx_V2) (left) and InfectedEx compounds (T_InfectedEx_All and T_InfectedEx_V2) (right). Error bars represent standard deviations. Asterisks indicate significance: ***p-value < 0.001 based on two-sided *t*-test (n = 16).

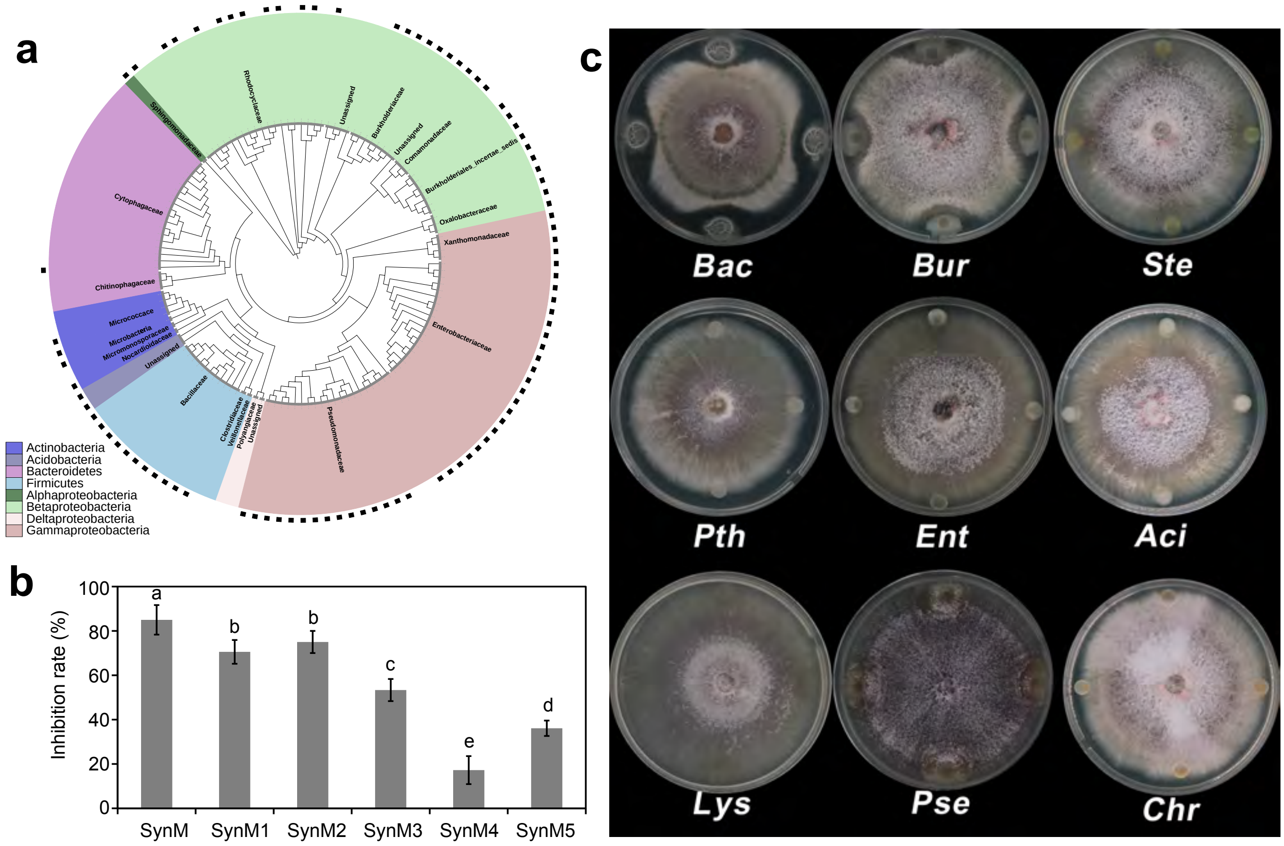

Fig. S5. Synthetic microbiota (SynM) construction. a, Culturable bacteria of the *Fusarium*-suppressed rhizosphere bacterial community. The inner ring represents the rhizosphere bacterial ASVs detected with a relative abundance greater than 0.05% in all of the *Fusarium*-suppressed rhizosphere bacterial communities. The outer ring with black squares represents ASVs that were identified in the cultivated bacteria derived from *Fusarium*-suppressed seedlings. **b**, The seed-borne *Fusarium* inhibition rate of the 11-strain SynM (constructed by 11 keystone ASVs) and multiple SynM controls (see details in **Table S4**). Error bars represent standard deviations (n = 3). Different letters above bars indicate significant differences (p-value < 0.05) according to Duncan's multiple comparison. **c**, The direct antagonistic activity of the 9 detected rhizosphere bacterial strains (ASVs) [*B. amyloliquefaciens* (*Bac*, left-top), *B. cenocepacia* (*Bur*, middle-top), *S. maltophilia* (*Ste*, right-top), *P. japonensis* (*Pth*, left-middle), *E. ludwigii* (*Ent*, middle-middle), *A. baumannii* (*Aci*, right-middle), *L. soli* (*Lys*, left-bottom), *P. stutzeri* (*Pse*, middle-bottom) and *C. rhizoplanae* (*Chr*, right-bottom)] against *Fusarium* using a inhibition zone assay.

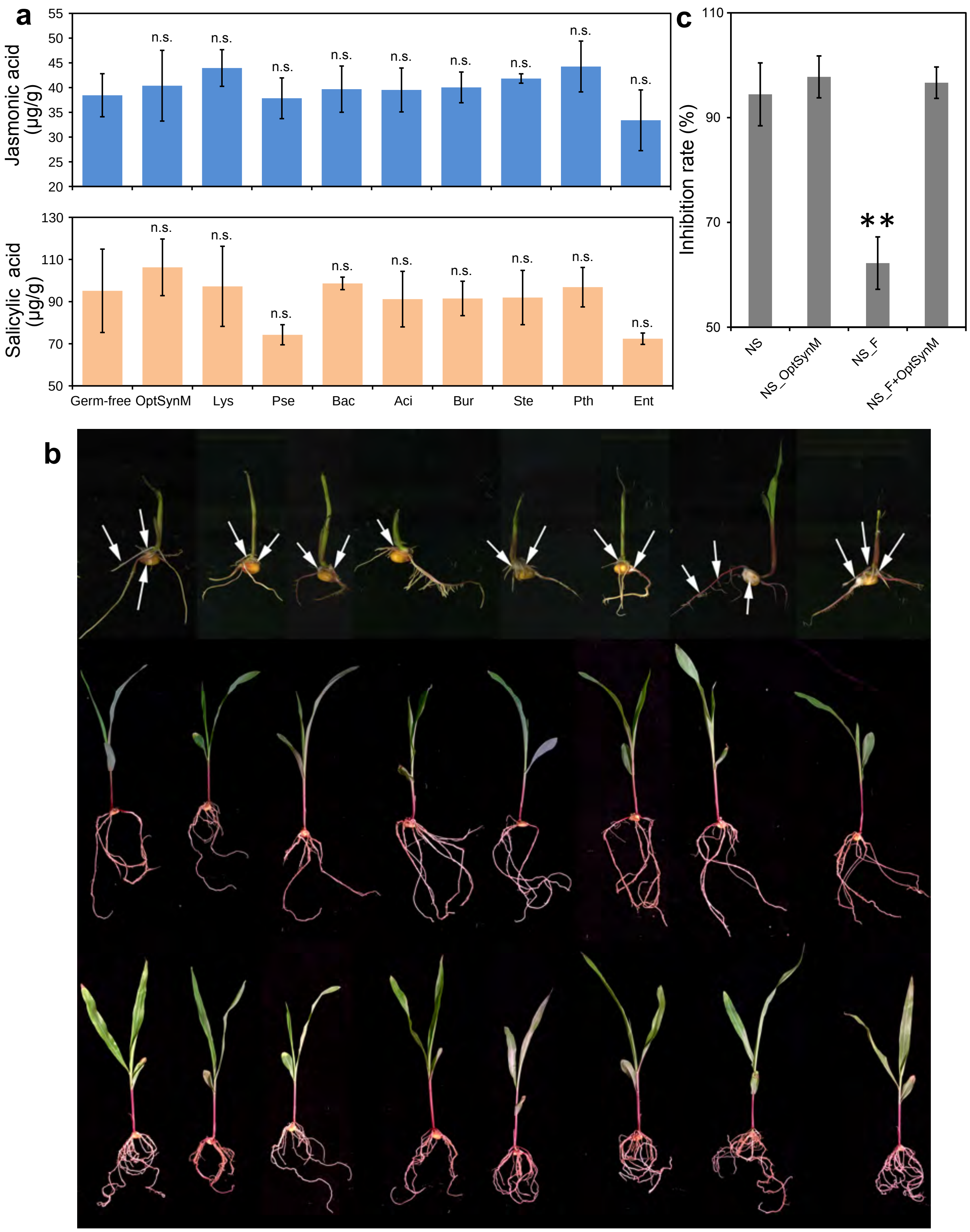

Fig. S6. Synthetic microbiota affects maize growth. a, The concentration of jasmonic acid and salicylic acid in plant tissue under the inoculation of 8-strain OptSynM and individual strains. Germ-free represents the non-inoculated treatment. Error bars represent standard deviations (n = 4). n.s: not significant between the inoculated treatment and germ-free treatment based on two-sided *t*-test. **b**, The plant morphology of eight maize cultivars when cultivated in sterile soil (top) under the inoculation of 8-strain OptSynM sterile soil (Middle) and non-sterile soil (bottom). Arrows point out the mycelium of seed-borne *Fusarium*. **c**, The seed-borne *Fusarium* inhibition rate under natural soil conditions. NS: non-sterile soil. NS_OptSynM: non-sterile soil inoculated with OptSynM. NS_F: non-sterile soil inoculated with *Fusarium*. NS_F+OptSynM: non-sterile soil inoculated with *Fusarium* and OptSynM. Asterisks indicate significance: **p value < 0.01 based on a two-sided *t*-test between NS and NS_F.

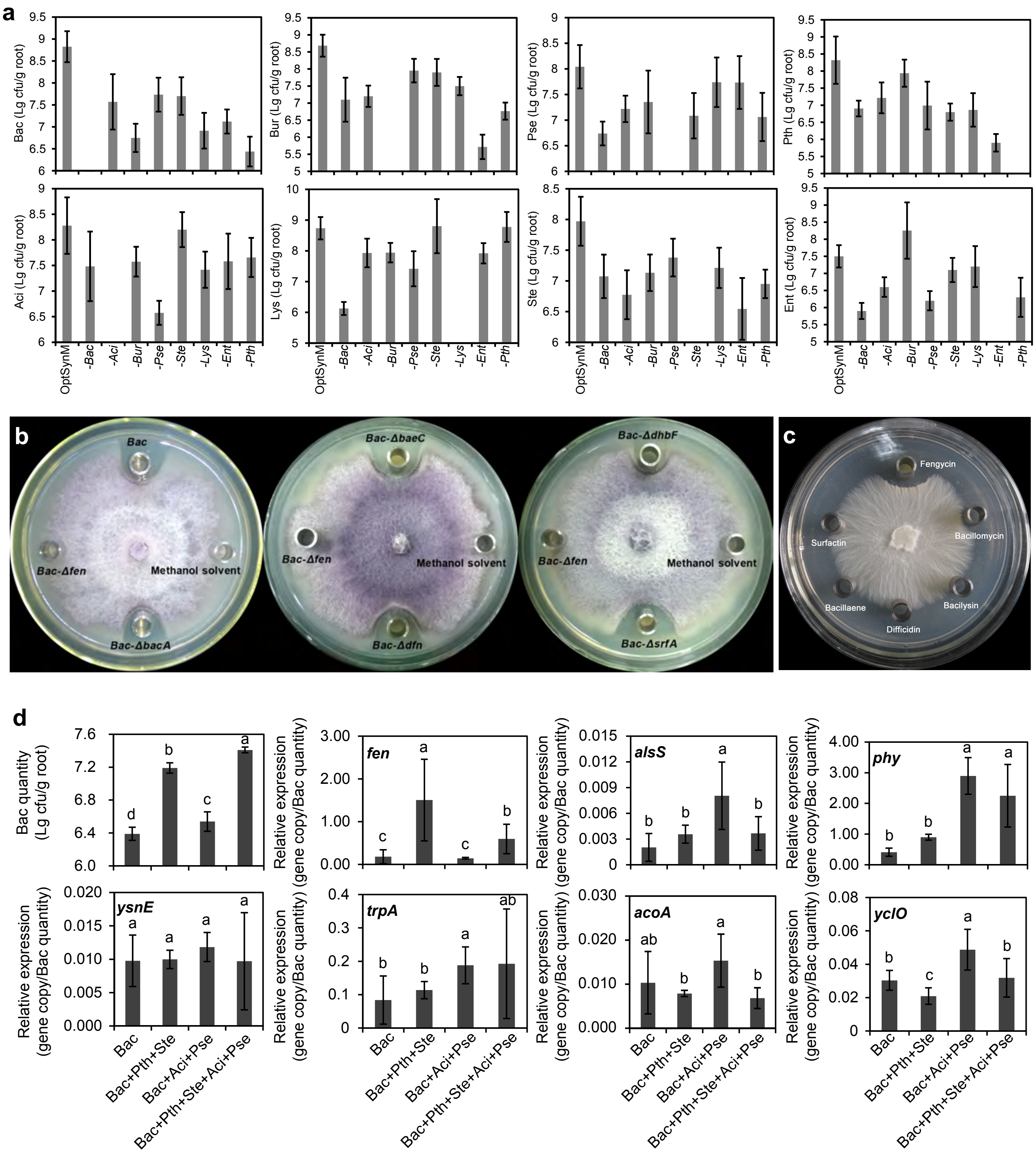

Fig. S7. Cooperative pattern within the 8-strain OptSynM. a, The colonization quantity of the 8-strain OptSynM and “one strain-knockout” OptSynMs in the rhizosphere. Error bars represent standard deviations (n = 4). **b**, The antagonistic activity of the mutants of *Bac* against *Fusarium* using inhibition zone assay. *Bac-Δfen*, *Bac-ΔbacA*, *Bac-Δdfn*, *Bac-ΔdhbF*, *Bac-ΔbaeC* and *Bac-ΔsrfA* represent the supernatant (in Oxford cup) of *Bac* mutants after knocking out the antagonistic *fen*, *bacA*, *dfn*, *dhbF*, *baeC* and *srfA* genes, respectively. *Bac* and methanol served as positive and negative controls, respectively. **c**, The antagonistic activity of the antagonistic gene-encoded products. The genes and products are listed in **Table S6**. **d**, *Bac* colonization quantity and the relative expression of the antagonistic related *fen* gene and six plant-growth-promoting related genes in the *Bac* strain in planta under inoculation with different strain combinations. The strain combinations are listed in **Table S7**. Error bars represent standard deviations (n = 6). Different letters above bars indicate significant differences (p-value < 0.05) according to Duncan’s multiple comparison.

Supplementary Table 1. Detailed information on the eight commercial maize cultivars used in this study.

Variety	Abbreviation	Cultivation region
Denghai605	DHA	East China
Longping206	LP	East China
Zhengdan958	ZDN	North China
Jingke968	JKE	North China
Fengtian843	FT	North China
Lianchuang808	LCH	Northeast China
Yuyu22	YYU	Northwest China
Zhengda999	ZDA	South China

Supplementary Table 2. The relative abundance of root exudate of Fusarium-infected (InfectedEx from sterilized soil) and Fusarium-suppressed (SuppressedEx from non-sterilized soil) seedlings at the one-leaf (V1), two-leaf (V2) and three-leaf (V3) stages.

	SuppressedEx_V1	SuppressedEx_V2	SuppressedEx_V3	InfectedEx_V1	InfectedEx_V2	InfectedEx_V3	Turkey's HSD test p-value (between SuppressedEx and InfectedEx)
Sugars	37.68±6.19	35.53±3.18	16.29±0.87	26.28±0.74	5.27±0.96	13.15±5.6	0.008
Organic acids	9.68±1.73	12.54±1.50	9.91±2.22	9.68±1.39	17.31±2.69	12.34±0.59	0.033
Alcohols	9.61±1.74	8.14±1.45	14.52±0.83	16.86±2.11	15.89±0.66	16.82±1.87	0.0004
Amines	0.68±0.05	0.85±0.16	1.37±0.19	1.34±0.23	1.16±0.03	1.33±0.13	0.036
Amino acids	0.51±0.05	0.80±0.09	0.67±0.09	0.99±0.22	0.84±0.13	0.56±0.05	0.222
Ketones	0.37±0.19	0.46±0.16	0.74±0.03	0.38±0.11	0.37±0.04	0.36±0.03	0.069
Esters	0.19±0.07	0.43±0.06	0.55±0.08	0.10±0.04	0.34±0.06	0.13±0.02	0.012
Phenols	0.07±0.01	0.11±0.01	0.12±0.03	0.09±0.02	0.10±0.02	0.13±0.01	0.561
Aldehydes	0.02±0.01	0.02±0.01	0.02±0.02	0.03±0.01	0.03±0.01	0.03±0.01	0.006
Others	7.63±0.44	9.19±1.02	13.30±0.34	6.86±0.37	7.85±0.65	9.12±0.34	0.005
Unclassified	23.59±2.65	24.42±2.38	28.46±1.34	30.88±2.22	41.23±1.77	38.25±0.44	< 0.0001
Unknown	9.95±4.87	7.51±2.82	14.05±2.65	6.50±0.52	16.28±1.89	9.79±4.03	0.885

Supplementary Table 3. The mixture of typical compounds used for soil incubation and chemotaxis assays.

	Mixture	Typical compounds
InfectedEx compounds	Typical compounds of InfectedEx in V2 stage (T_InfectedEx_V2)	2-Ketocaproic acid, mannitol, L-Malic acid
	Typical compounds of InfectedEx in all stages (T_InfectedEx_All)	2-Ketocaproic acid, mannitol, L-Malic acid, sulfuric acid, glucose
SuppressedEx compounds	Typical compounds of SuppressedEx in V2 stage (T_SuppressedEx_V2)	melibiose, allose, sucrose
	Typical compounds of SuppressedEx in all stages (T_SuppressedEx_All)	xylose, palmitic acid, melibiose, allose, sucrose, threonic acid

Supplementary Table 4. Composition of synthetic microbiota at the ASV level of taxonomic resolution.

Core ASVs	Different ASVs from the same genera of the core ASVs		Enriched ASVs in *Fusarium*-suppressed seedling	Depleted ASVs in *Fusarium*-suppressed seedling	Completely randomly selected ASVs
SynM	SynM1	SynM2	SynM3	SynM4	SynM5
ASV_55_g_*Pseudomonas* (*Pse*)	ASV_81_g_*Pseudomonas*	ASV_90_g_*Pseudomonas*	ASV_215_g_*Burkholderia*	ASV_2397_g_*Chitinophaga*	ASV_60_g_*Klebsiella*
ASV_96_g_*Bacillus* (*Bac*)	ASV_430_g_*Bacillus*	ASV_296_g_*Bacillus*	ASV_322_g_*Azotobacter*	ASV_3443_g_*Promicromonospora*	ASV_730_g_*Flavobacteria*
ASV_138_g_*Lysobacter* (*Lys*)	ASV_405_g_*Lysobacter*	ASV_382_g_*Lysobacter*	ASV_456_g_*Azospirillum*	ASV_3983_g_*Lysinibacillus*	ASV_2651_g_*Amimobacter*
ASV_158_g_*Burkholderia* (*Bur*)	ASV_215_g_*Burkholderia*	ASV_309_g_*Burkholderia*	ASV_367_g_*Pseudoxanthomonas*	ASV_624_g_*Streptophyta*	ASV_3983_g_*Lysinibacillus*
ASV_221_g_*Enterobacter* (*Ent*)	ASV_356_g_*Enterobacter*	ASV_630_g_*Enterobacter*	ASV_250_g_*Lentzea*	ASV_1642_g_*Massilia*	ASV_441_g_*Agromyces*
ASV_816_g_*Pseudoxanthomonas* (*Pth*)	ASV_116_g_*Pseudoxanthomonas*	ASV_501_g_*Pseudoxanthomonas*	ASV_88_g_*Pontibacter*	ASV_1241_g_*Kosakonia*	ASV_573_g_*Dyadobacter*
ASV_1383_g_*Stenotrophomonas* (*Ste*)	ASV_459_g_*Stenotrophomonas*	ASV_643_g_*Stenotrophomonas*	ASV_392_g_*Enterobacter*	ASV_706_g_*Methyloversatilis*	ASV_2434_g_*Microbacterium*
ASV_1649_g_*Acinetobacter* (*Aci*)	ASV_324_g_*Acinetobacter*	ASV_1028_g_*Acinetobacter*	ASV_1268_g_*Ensifer*	ASV_166_g_*Fictibacillus*	ASV_82_g_*Streptomyces*
ASV_2096_g_*Chryseobacterium* (*Chr*)	ASV_951_g_*Chryseobacterium*	ASV_535_g_*Chryseobacterium*	ASV_2373_g_*Devosia*	ASV_32_g_*Streptophyta*	ASV_1683_g_*Comamonas*
ASV_196_g_*Pseudomonas* (*Pse2*)	ASV_26_g_*Pseudomonas*	ASV_397_g_*Pseudomonas*	ASV_41_g_*Agromyces*	ASV_92_g_*Streptomyces*	ASV_3671_g_*Pseudodubacter*
ASV_971_g_*Pantoea* (*Pan*)	ASV_1152_g_*Pantoea*	ASV_1638_g_*Pantoea*	ASV_934_g_*Agrobacterium*	ASV_335_g_*Kaistia*	ASV_205_g_*Niastella*

Supplementary Table 5. The specific primer set for each strain of the 8-strain OptSynM.

Strain	Primers	Sequence (5'-3')	Length
Lys	Forward	TGGCGAAGGTAAGAATCAG	134
	Reverse	GACCGAAGCAGTCAAGTT	
Pse	Forward	GTGCTGGTCGCTTATCTG	187
	Reverse	CGGCAACCTCTATCTGTTC	
Pth	Forward	GAGCCTTGCGTTCATAGA	179
	Reverse	CCAGATAGCCTTCCATTCC	
Ste	Forward	GACGCCTCCATCAATCAA	189
	Reverse	CACCATTGCCATCTGAATAG	
Bac	Forward	GTGTCAGCAGTTCATACCA	187
	Reverse	CCGATAGACAGAGAACCTTG	
Bur	Forward	GTGGTGGTCCAATTCCTATT	113
	Reverse	CTTCCTGCTGCTGTTGTT	
Aci	Forward	ATTTAGTATCTGGTGAAGTCATCCGTA	92
	Reverse	CCGACAAATAAAGCTTGAGTAACTCC	
Ent	Forward	CAGCAGGAGGTCATTCAT	113
	Reverse	CGGTGTACGAGGATATTGA	

Supplementary Table 6. The primers and products of the antagonistic and plant-growth-promoting related genes in the *Bac* strain.

Gene	Product	Primers	Sequence (5'-3')	Length
srfA	Surfactin	srfAC-F	CCGCAAACCTTTACTT	200
		srfAC-R	AGGATGTCGGACCAGA	
baeC	Bacillaene	baeC-F	CGCACGGATTACATAC	190
		baeC-R	AACTCTTGTTTCGCTTC	
fen	Fengycin	fen-F	AGCAAGGGAGACACGA	196
		fen-R	CGAGAACCTGGGAGAC	
dfn	Difficidin	dfn-F	GCTGCTGGCGGATAAA	129
		dfn-R	GGAAGATGGAATGTGGC	
bacA	Bacilysin	bacA-F	CTGAAGGGACAAGCAGTGAG	200
		bacA-R	GATAGGAGACGGGTGGGATA	
dhbF	Bacillibactin	dhbF-F	AGAGGTTTCGCTATTGG	273
		dhbF-R	TTCGGCTTGTATGTTCC	
ysnE	IAA	ysnE-F	GGCTGTGAACCTTTGC	261
		ysnE-R	CGGCTGTTTAGCTCCT	
trpA	Tryptophan	trpA-F	GACATAGACGGTCTGCT	150
		trpA-R	TCAAACGGTCTTCACTT	
alsS	2,3-Butanediol	alsS-F	ACCGTGCTATTATCCATC	150
		alsS-R	TGTCACAGCGTCGTGT	
acoA	Acetoin	acoA-F	CCAATAACCAAGGGAC	150
		acoA-R	GAATCACAGGCTGACG	
phy	Phytase	phy-F	AAATCAGGCTTAGTCGT	150
		phy-R	AATGGCGTAAATCTCAA	
yclO	Ferrichrome	yclO-F	ATCTTAATTTCGTCATCTCC	150
		yclO-R	TGTTGAACAGCGTGCC	

Supplementary Table 7. Strain combinations based on their functional intensification roles on *Bac*.

Functional intensification role	Strains
Single strain	*Bac*
Antagonistic (Antag) function intensification	*Bac*+*Pth*+*Ste*
Plant-growth-promoting (PGP) function intensification	*Bac*+*Aci*+*Pse*
Antag and PGP functions intensification	*Bac*+*Pth*+*Ste*+*Aci*+*Pse*
